# Parkinson’s disease assessment with selfie video via feature engineering and explainable AI

**DOI:** 10.1371/journal.pone.0357902

**Published:** 2026-09-22

**Authors:** Sitthatka Jaratsaeng, Anchalee Techasen, Narongrit Kasemsap, Thanapong Intharah

**Affiliations:** 1 Visual Intelligence Laboratory, Faculty of Science, Khon Kaen University, Khon Kaen, Thailand; 2 Faculty of Associated Medical Sciences, Khon Kaen University, Khon Kaen, Thailand; 3 Department of Medicine, Faculty of Medicine, Khon Kaen University, Khon Kaen, Thailand; Middle Technical University, IRAQ

## Abstract

Parkinson’s disease (PD) is a neurodegenerative disorder fundamentally characterized by motor impairments and diminished facial expressivity. This study investigates the efficacy of feature engineering approaches applied to facial video data captured via smartphone frontal cameras to predict PD presence, disease severity, and motor function scores. Within a cohort of 40 participants, a combination of three predictive models, four facial movement scenarios, and five frame selection strategies was rigorously evaluated. Regarding PD classification, the XGBoost model achieved maximum performance using the smiling video scenario with frame skipping combined with PCA retaining 95% of the variance, yielding an accuracy of 0.77, a precision of 0.75, a recall of 0.86, and an F1-score of 0.79. For Hoehn & Yahr severity estimation, the Random Forest model implemented with the smiling video, frame skipping, and no PCA attained the best result, with the lowest RMSE of 1.30 and a MAPE of 38.36%. Furthermore, in The Movement Disorder Society-sponsored revision of the Unified Parkinson’s Disease Rating Scale (MDS-UPDRS) Part III motor score estimation, the XGBoost model outperformed other configurations using the smiling video with 3-frame averaging and PCA retaining 95% of the variance, achieving an RMSE of 19.34 and a MAPE of 45.33%. Crucially, the Explainable AI analysis identified that lip and mouth corner movements were the most critical features for both PD classification and Hoehn & Yahr estimation. In contrast, eyebrow and eye movements were the dominant features for MDS-UPDRS Part III motor score estimation. These findings indicate that facial dynamics carry information relevant to PD assessment and support further development of non-invasive, accessible screening tools, although the accuracy achieved here is not yet sufficient to substitute for clinical evaluation.

## Introduction

Parkinson’s disease is a complex and distinctive neurological disorder, primarily characterized by the slowing down of body movements due to the loss of dopaminergic neurons in the midbrain region known as the substantia nigra. However, the exact cause of Parkinson’s disease remains unclear, and its early-stage diagnosis is challenging for physicians since initial symptoms are often subtle and vary among individuals [1]. Patients may exhibit different symptoms depending on the extent of brain and nervous system damage.

The disease was first systematically described in 1817 by James Parkinson in *An Essay on the Shaking Palsy*, providing the earliest clinical characterization of what is now known as Parkinson’s disease [2]. Subsequent research in the mid-20th century by Arvid Carlsson demonstrated the crucial role of dopamine in motor control and linked dopamine deficiency to the motor symptoms of PD [3]. This discovery formed the foundation for dopaminergic replacement therapies, such as L-DOPA, which remain the cornerstone of PD treatment today. In addition, advancements in genetic studies have revealed that mutations in several genes are associated with familial and early-onset forms of Parkinson’s disease, providing deeper insight into its molecular and hereditary mechanisms [4]. Subsequently, Jean-Martin Charcot, a French neurologist, expanded the understanding of the disease by introducing the term “Parkinson’s disease” in place of “shaking palsy” and by developing clinical classification criteria for diagnosis [5].

One of the hallmark symptoms of Parkinson’s disease is the loss of facial expressivity, known as hypomimia, resulting from the degeneration of neurons in the basal ganglia and substantia nigra, which are responsible for controlling facial muscles. Consequently, patients exhibit reduced or slowed facial movements and diminished ability to express emotions naturally [6]. Hypomimia not only affects patients’ communication and quality of life but also serves as a clinical indicator correlated with disease severity, making facial analysis an essential approach for studying and monitoring the disease [7].

To systematically evaluate and monitor Parkinson’s disease severity, several clinical assessment tools have been developed. One of the most widely used tools is the Hoehn & Yahr scale, proposed by Hoehn and Yahr [8], which categorizes the disease into five stages from early-stage (Stage 1), where symptoms occur on one side of the body, to the most severe stage (Stage 5), where patients are wheelchair-bound or unable to move independently. This scale provides a standardized method for clinicians to track disease progression and evaluate treatment effectiveness.

A more comprehensive instrument was later introduced by Goetz et al. [9]: the Movement Disorder Society-sponsored revision of the Unified Parkinson’s Disease Rating Scale (MDS-UPDRS), an improved version of the original UPDRS. It consists of four main parts: Non-motor aspects of daily living, Motor experiences of daily living, Motor examination, and Motor complications. Among these, Part III: Motor Examination has received significant research attention as it reflects the severity of motor symptoms and serves as a key indicator for diagnosis and patient follow-up.

Detecting hypomimia by the naked eye is difficult, especially in the early stages of the disease, which has led to growing research interest in facial analysis to assist in diagnosis and monitoring patient progression [7].

In addition to distinguishing patients from non-patients, assessing the severity of Parkinson’s disease is another essential aspect of research. The ability to predict Parkinson’s disease severity and motor scores is crucial for patient management, helping clinicians design personalized treatment plans, including medication selection and dosage adjustment, physical rehabilitation, and ongoing progress tracking. Moreover, predicting disease severity is valuable for evaluating treatment effectiveness and monitoring long-term disease progression, allowing rapid adaptation of therapeutic strategies when patient conditions change [10].

This research focuses on the development of a facial video analysis system for Parkinson’s disease patients, in which OpenFace 2.0 is used to generate facial action timelines that serve as input features for machine learning (ML) models. Two complementary objectives are thereby enabled:

1. **Classification:** To distinguish between Parkinson’s patients and normal people, serving as an accurate tool for preliminary diagnosis.
2. **Regression:** To estimate the Hoehn & Yahr scale and MDS-UPDRS Part III score for each patient, enabling continuous assessment of disease severity and progression.

Such models can benefit both research and clinical applications. By integrating facial and video data with AI and machine learning, the model can extract critical movement-related features that enhance clinical decision-making with evidence-based support. Furthermore, feature importance was analyzed to identify which facial movement patterns most influence severity assessments and motor scores, offering potential insights for improving future patient evaluations.

In summary, this research aims to develop a system for analyzing and predicting Parkinson’s disease severity from facial videos, supporting both patient classification and continuous severity assessment. The study integrates face detection (face landmark), video analysis (OpenFace 2.0), and machine learning to create a valuable clinical tool for both physicians and patients.

This paper extends our earlier conference proceeding work [11], introducing a regression model for severity scoring and Explainable AI for clinical interpretability, alongside a more robust experimental evaluation. In this work, the complete workflow is described, starting from smartphone front-camera videos and including the design of facial action scenarios and data-processing methods. The primary contributions of this research are summarized as follows:

1. A systematic feature engineering framework comprising five temporal pre-processing strategies for facial-based PD classification, extending the preliminary methodologies of the previous conference work [11].
2. The introduction of a regression-based framework to quantify PD severity, specifically estimating the Hoehn & Yahr scale and MDS-UPDRS Part III motor scores.
3. The implementation of Explainable AI using PCA back-mapping to identify the specific facial landmark drivers such as lip and mouth dynamics that influence each predictive task.
4. A clinical contextualization of the model’s performance, evaluating prediction errors against clinical standards to assess its practical utility as a non-invasive assessment tool.

## Related work

One of the tools used for facial analysis is face landmark, which enables the detection of facial movements, expressions, and gestures in detail. OpenFace 2.0 [12] extends this capability by detecting faces, head pose, eye gaze, and upper-body movements in real time, making it a potential tool for assessing long-term motor symptoms. It builds on Dlib, an open-source library developed by King [13], which provides efficient face detection and landmark localization based on Histogram of Oriented Gradients (HOG). OpenFace integrates Deep Convolutional Neural Networks (DCNNs) to extract 2D and 3D facial landmarks, eye gaze, and facial action units (AUs) accurately, even in real time, offering valuable tools for analyzing subtle motor impairments in Parkinson’s disease.

OpenFace was introduced in 2016 by Baltrušaitis et al. [14] as an open-source toolkit for facial landmark detection, head pose estimation, and expression analysis. It employs DCNNs trained on large-scale facial datasets, enabling robust face and landmark detection under various poses and lighting conditions [14]. Later, OpenFace 2.0 was introduced as an enhanced version with improved landmark accuracy, real-time performance, and expanded functionalities for analyzing facial emotions and behaviors in greater detail. OpenFace 2.0 can process both images and videos by segmenting videos into frames (approximately three frames per 0.1 seconds). Each frame is recorded with parameters such as frame ID, timestamp, confidence, and success rate to ensure detection reliability. The facial landmarking module in OpenFace 2.0 tracks 68 facial points in both 2D and 3D, covering key facial structures such as eyes, nose, mouth, and facial contours [12]. Additionally, OpenFace 2.0 extracts head pose, eye gaze, and Facial Action Units (AUs) both rigid (e.g., head rotation or tilt) and non-rigid (e.g., smiling, frowning, mouth opening). The intensity and presence of each AU are recorded on a numerical scale (0–5), along with face size, position, and rotation parameters [12]. This tool enables detailed and continuous analysis of facial expressions and motor symptoms in Parkinson’s patients, supporting research on patient classification, disease severity estimation, and tracking of motor symptom progression through landmark and AU data extracted from facial videos [12]. Parkinson’s disease, caused by dopaminergic neuron degeneration in the substantia nigra, leads to tremors, rigidity, bradykinesia, and reduced facial expressivity (hypomimia), which substantially affects patients’ quality of life [1,3].

A study by Bandini, A., et al. [15] demonstrated that facial motion analysis can effectively distinguish Parkinson’s patients from healthy controls, particularly when using facial landmark detection technology, which quantitatively measures facial muscle movements. Therefore, facial features have emerged as potential clinical indicators for evaluating Parkinson’s severity alongside standard criteria such as the Hoehn & Yahr scale and MDS-UPDRS. Rajnoha et al. [16] proposed a facial video-based Parkinson’s classification method using face landmark techniques that mapped 68 facial points to calculate motion embeddings extracted by a Convolutional Neural Network (CNN), which were then classified using K-Nearest Neighbors (KNN), XGBoost, Decision Tree, Random Forest, and Support Vector Machine (SVM) with cross-validation. Among these classifiers, the Decision Tree algorithm achieved the highest classification accuracy of 67.33%. Building upon this, Hou et al. [17] developed a multi-feature approach using three types of features: geometric, texture, and their combination. The study revealed significant facial motion differences between Parkinson’s and control groups, particularly around the mouth corners, indicative of hypomimia. Among the tested models (KNN, Random Forest, and SVM), the SVM with combined geometric and texture features yielded the best performance, achieving an accuracy of 0.94 and an F1-score of 0.94, demonstrating its high efficiency in Parkinson’s classification. While earlier research mainly focused on classification, other studies have explored disease severity estimation and clinical score estimation, which are essential for tracking disease progression and treatment planning. Jackson et al. [10] investigated factors predicting Parkinson’s disease progression, particularly motor function, using Hoehn & Yahr stages in combination with Dopamine Transporter Single-Photon Emission Computed Tomography (DAT-SPECT) imaging. Results showed a significant correlation between striatal uptake levels and Hoehn & Yahr stages, indicating that neuroimaging biomarkers and clinical scales can complement each other in assessing disease severity. Similarly, Ma et al. [18] proposed a predictive approach for MDS-UPDRS Motor Scores, comparing Multiple Linear Regression with machine learning models such as Random Forest, Support Vector Regression (SVR), and Gradient Boosting. Their results demonstrated that machine learning-based models outperformed linear regression, with the Random Forest providing the most accurate predictions and lowest error rates, underscoring the potential of machine learning in early-stage Parkinson’s progression prediction.

Recent research has made substantial progress in facial-expression analysis for PD, particularly with larger datasets, temporal modeling, and multi-modal approaches. For example, the study by Adnan et al. [19] collected facial-expression videos from 1,059 participants (256 with PD) and achieved an accuracy of approximately 87.90% using an ensemble of machine-learning models. Their results demonstrate the feasibility of large-scale, low-cost PD screening from simple webcam-captured expressions.

More recently, Duan et al. introduced QAFE-Net [20], which focuses on expression-quality regression rather than binary detection. Using the PFED5 dataset, containing five types of facial expressions with per-expression MDS-UPDRS severity labels, QAFE-Net models temporal landmark heatmaps with a transformer-based architecture. This approach provides more fine-grained severity estimation and illustrates the importance of temporal cues rather than static frame features.

In addition, multi-modal systems have shown strong performance for at-home PD screening. Islam et al. [21] proposed UFNet, which integrates facial videos, speech, and motor-task inputs using uncertainty-aware fusion. Their findings indicate substantially higher accuracy and AUROC compared with facial-only pipelines, highlighting the limitations of single-modality approaches.

A recent systematic review by Di Biase et al. [22] also confirms that video-based, markerless computer-vision systems can approximate clinician ratings for diagnostic and severity assessment tasks. However, the review emphasizes the need for standardized protocols, temporal modeling, and cross-setting validation to ensure reliability.

While recent studies have demonstrated the potential of deep learning architectures such as CNNs, LSTMs, and Transformers for temporal facial analysis [19–21], these approaches typically require substantially larger datasets to achieve robust generalization. While the studies reviewed above primarily apply explainability techniques within a computer vision and facial landmark-based context, XAI has also been extended to other clinical dimensions of Parkinson’s disease. For instance, Colautti et al. [23] applied artificial neural networks combined with explainable AI techniques to predict cognitive decline in Parkinson’s disease patients, demonstrating that XAI can provide interpretable insights beyond motor symptom analysis. This broader application of XAI across both motor and non-motor dimensions of the disease underscores its growing role as a critical tool for enhancing the transparency and clinical relevance of machine learning-based assessment methods. Given the sample size of 40 participants, classical machine learning models (SVM, Random Forest, XGBoost) combined with carefully engineered handcrafted features from OpenFace 2.0 were used for the PD analysis tasks. This decision was motivated by three factors: (1) reduced risk of overfitting with limited training data, (2) enhanced interpretability through direct feature importance extraction, and (3) computational efficiency suitable for potential deployment on resource-constrained devices.

## Materials and methods

**Fig 1:**
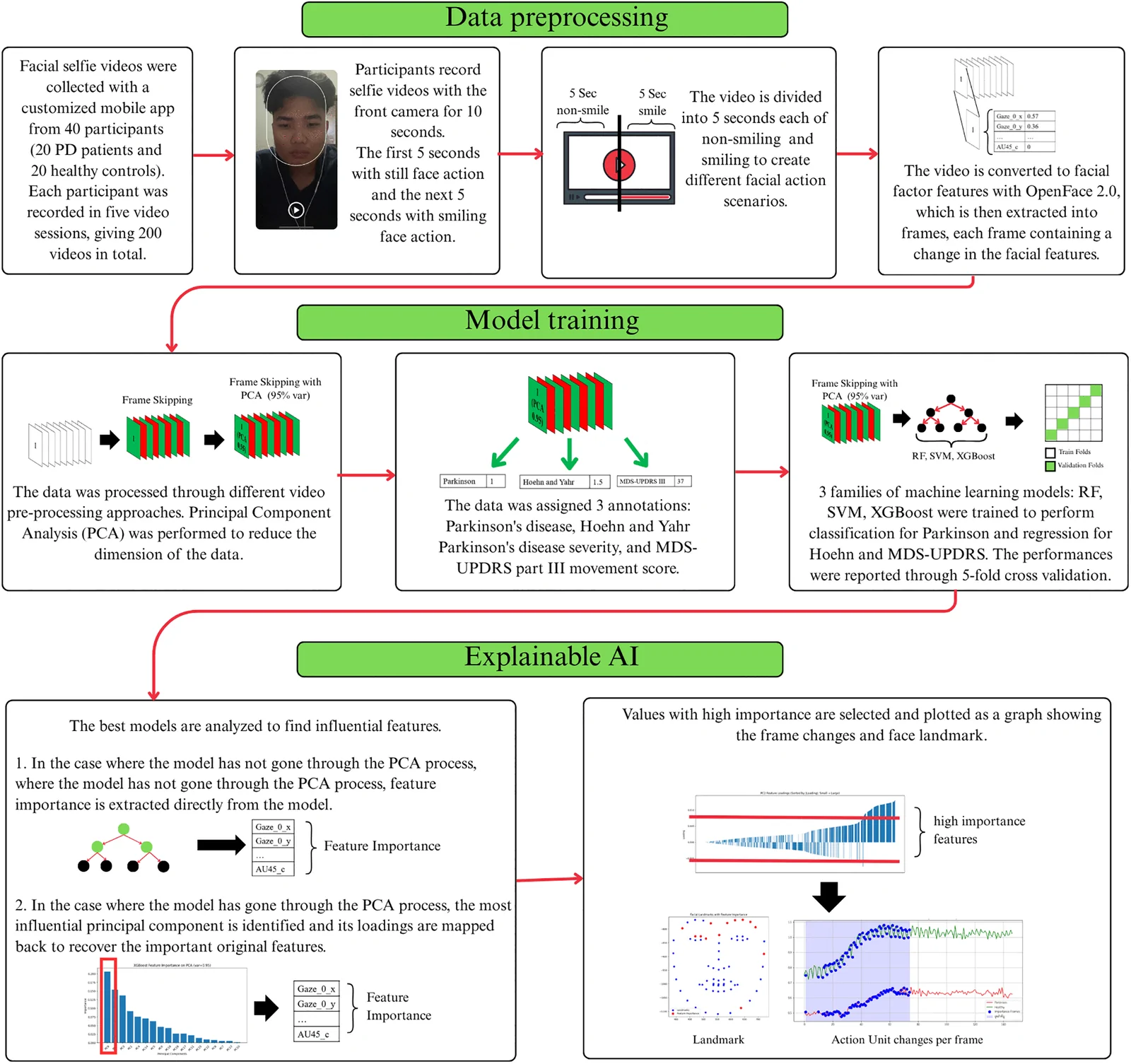
Overview of the proposed method for Parkinson’s disease assessment from facial video. (The individual in this manuscript has given written informed consent (as outlined in PLOS consent form) to publish these case details.).

To investigate the feasibility of classifying and predicting the severity of PD through facial video analysis, this study collected selfie videos from PD patients and healthy controls to perform classification and disease severity estimation using machine learning techniques. The videos were divided into multiple facial action scenarios to capture facial movement patterns across different intervals. OpenFace 2.0 [12] was used to detect and extract facial features, including Facial Landmarks, Action Unit (AU) Intensities, and Head Pose, reflecting differences in facial expressions between patients and controls. Extracted data were processed using five data processing methods: sequential frames, frame skipping, frame averaging, 3-frame averaging, and frame differencing, to determine which method yielded the most effective results for PD classification. As part of the feature engineering process, Principal Component Analysis (PCA) was subsequently applied for dimensionality reduction, retaining principal components with cumulative explained variance of 0.95 and 0.98 [24]. The reduced data were then used to train ML models, including Support Vector Machine (SVM) [25], Random Forest (RF) [26], and Extreme Gradient Boosting (XGBoost) [27]. Model performance was evaluated using k-fold cross-validation [28], with metrics including accuracy, precision, recall, and F1-score for classification, and Root Mean Squared Error (RMSE) and Mean Absolute Percentage Error (MAPE) for regression of Hoehn & Yahr [8] and MDS-UPDRS Part III scores [9]. The entire workflow is illustrated in Fig 1.

### Participants and data collection

Data collection adhered to ethical standards for research involving human participants, and informed consent was obtained from all participants to ensure full understanding of the study objectives, procedures, and their right to withdraw at any time without consequences for medical care or personal rights. Facial videos were recorded from 40 participants, comprising 20 individuals diagnosed with PD and 20 healthy controls. A clinical specialist conducted the diagnostic confirmation for all PD participants before their inclusion in the study. The same specialist also supervised the data collection process to ensure consistent evaluation across all recording conditions. For control participants, who did not exhibit any PD symptoms, the Hoehn & Yahr stage and MDS-UPDRS motor scores were assigned a value of zero to reflect the absence of motor impairment for machine learning model development.

Each participant completed five video sessions, producing a total of 200 clips, each approximately 10 seconds in duration. Each clip was segmented into two parts: the first 5 seconds captured neutral facial behavior (still-face condition), and the subsequent 5 seconds recorded smiling facial actions (expressive condition). This structured protocol enabled detailed examination of both spontaneous and voluntary facial expressions while ensuring the protection of participant confidentiality and privacy throughout the study. The individual in this manuscript has given written informed consent (as outlined in PLOS consent form) to publish these case details. The structured dataset extracted from these recordings is archived at the Harvard Dataverse (https://doi.org/10.7910/DVN/CT34N0) [29].

### Ethics statement

The study protocol was approved by the Center for Ethics in Human Research (CEHR), Khon Kaen University (HE674035). The recruitment and data collection period for this study spanned from 01/05/2025–31/05/2025. Written informed consent was obtained from all participants prior to their inclusion in the study, strictly following the protocols approved by the Center for Ethics in Human Research, Khon Kaen University. The individual in this manuscript has given written informed consent (as outlined in PLOS consent form) to publish these case details.

### Facial action scenarios

All 200 videos were organized and analyzed according to different facial action scenarios to study the impact of facial behavior on PD classification and regression:

1 **Original** All 200 clips of 10 seconds each, without segmentation, suitable for overall facial behavior analysis.
2 **Non-smile** Using only the first 5 seconds of each clip (still face action) to focus on neutral facial movement analysis.
3 **Smile** Using only the last 5 seconds of each clip (smiling face action) to focus on facial muscle movements and emotional expressions.
4 **Combined** 400 clips combining Non-smile and Smile clips to increase data size and provide sufficient data for ML model training and evaluation.

Each scenario was processed using video processing methods to evaluate which facial scenario yielded the best performance in PD classification and severity estimation.

### Facial landmark detection (OpenFace 2.0)

**Fig 2:**
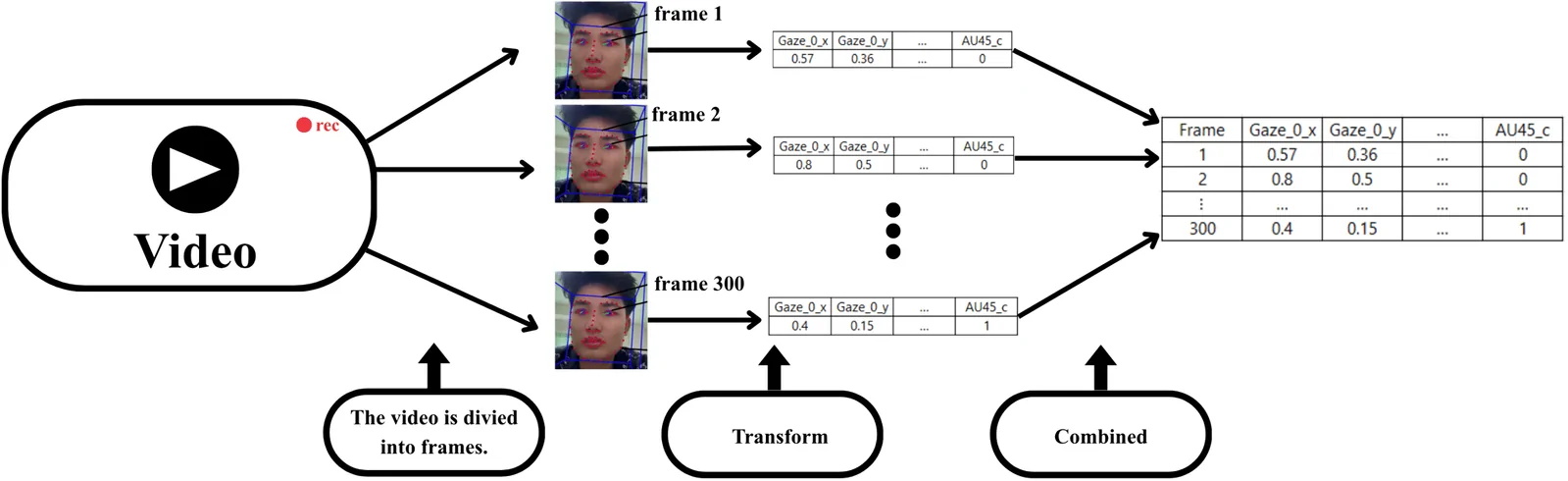
Feature preparation using OpenFace 2.0 (The individual in this manuscript has given written informed consent (as outlined in PLOS consent form) to publish these case details.).

Facial landmark detection used OpenFace 2.0 [12], an open-source tool for detailed facial analysis. It detects 68 facial landmarks and additional features such as head pose, eye gaze, and AUs in 2D and 3D. OpenFace 2.0 processed each video frame and extracted numerical features, resulting in a data table per video with 300 rows (frames) and 709 columns (features), as shown in Fig 2.

These features can be applied for PD classification and severity estimation. Previous studies [16,17] demonstrated that facial motion analysis is a crucial predictor for PD and can be used to predict MDS-UPDRS and Hoehn & Yahr scores.

### Feature engineering via data processing methods

**Fig 3:**
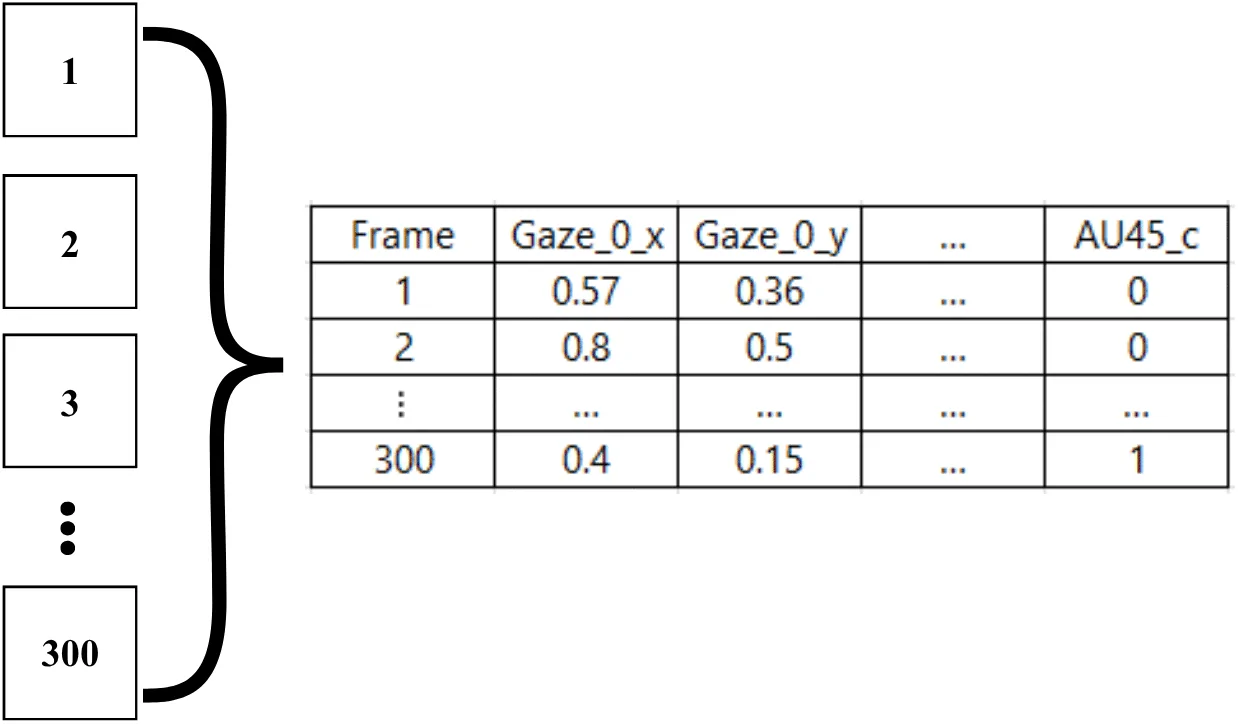
Sequential frames.

As a core component of the feature engineering pipeline, five pre-processing strategies were applied to prepare facial video data for ML modeling [12,17]:

**Sequential Frames** All 300 frames per video flattened into a single row (212,700 columns per participant) to preserve the entire facial movement sequence following Fig 3.

**Fig 4:**
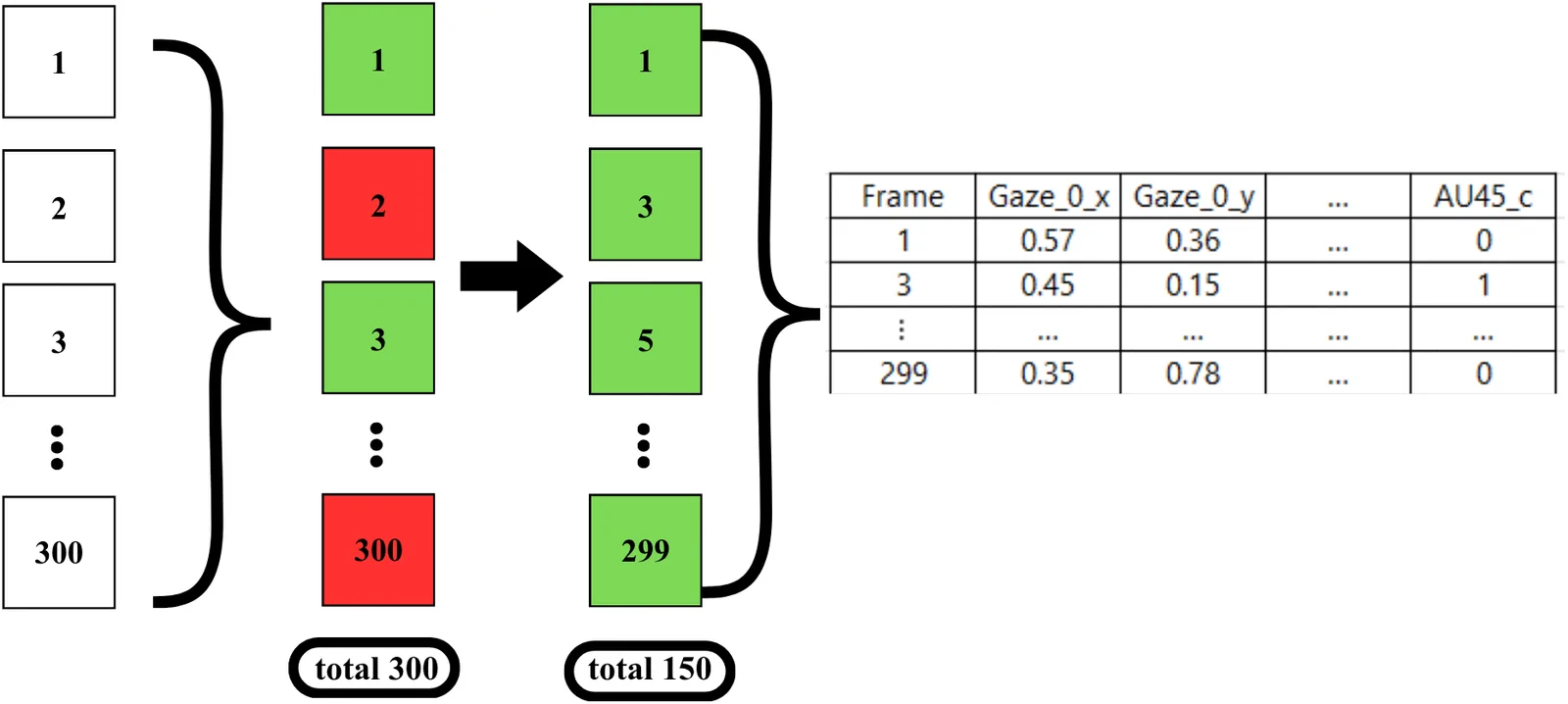
Frame skipping.

**Frame Skipping** Skipping every other row, reducing columns to 106,350 per participant, decreasing training time and redundancy following Fig 4.

**Fig 5:**
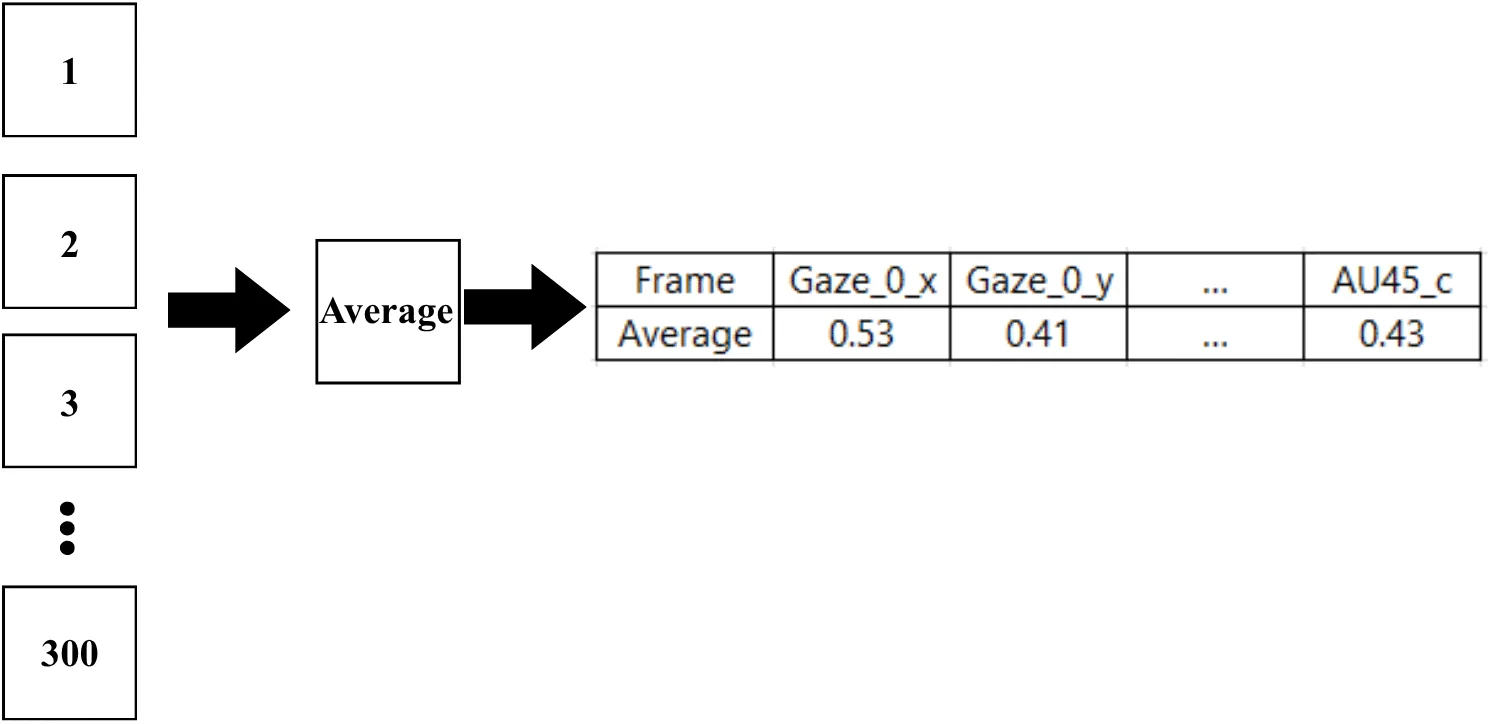
Frame averaging.

**Frame Averaging** Averaging all 300 frames to one row of 709 columns per video, reducing noise and emphasizing significant facial movements following Fig 5.

**Fig 6:**
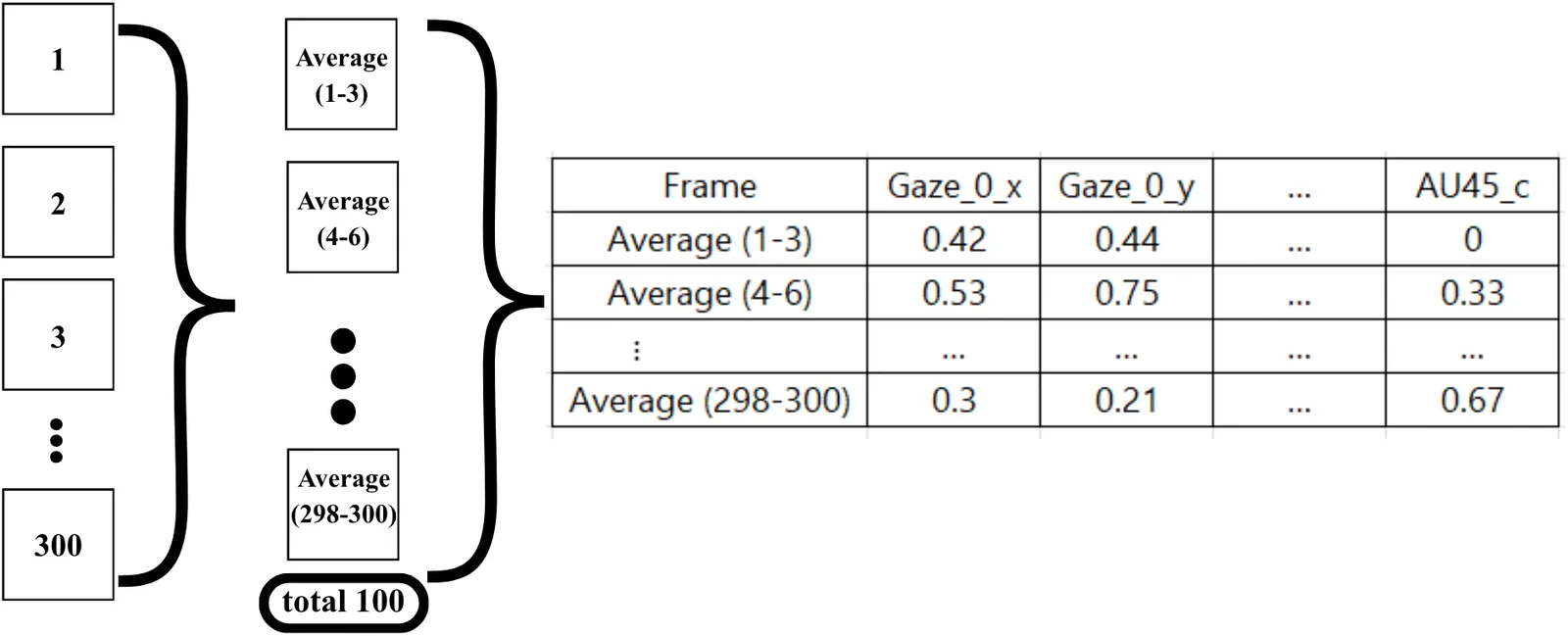
3-frame averaging.

**3-Frame Averaging** Averaging every three frames into 100 rows per video, then flattened to one row per participant (70,900 columns), preserving sequential movement while reducing dimensionality following Fig 6.

**Fig 7:**
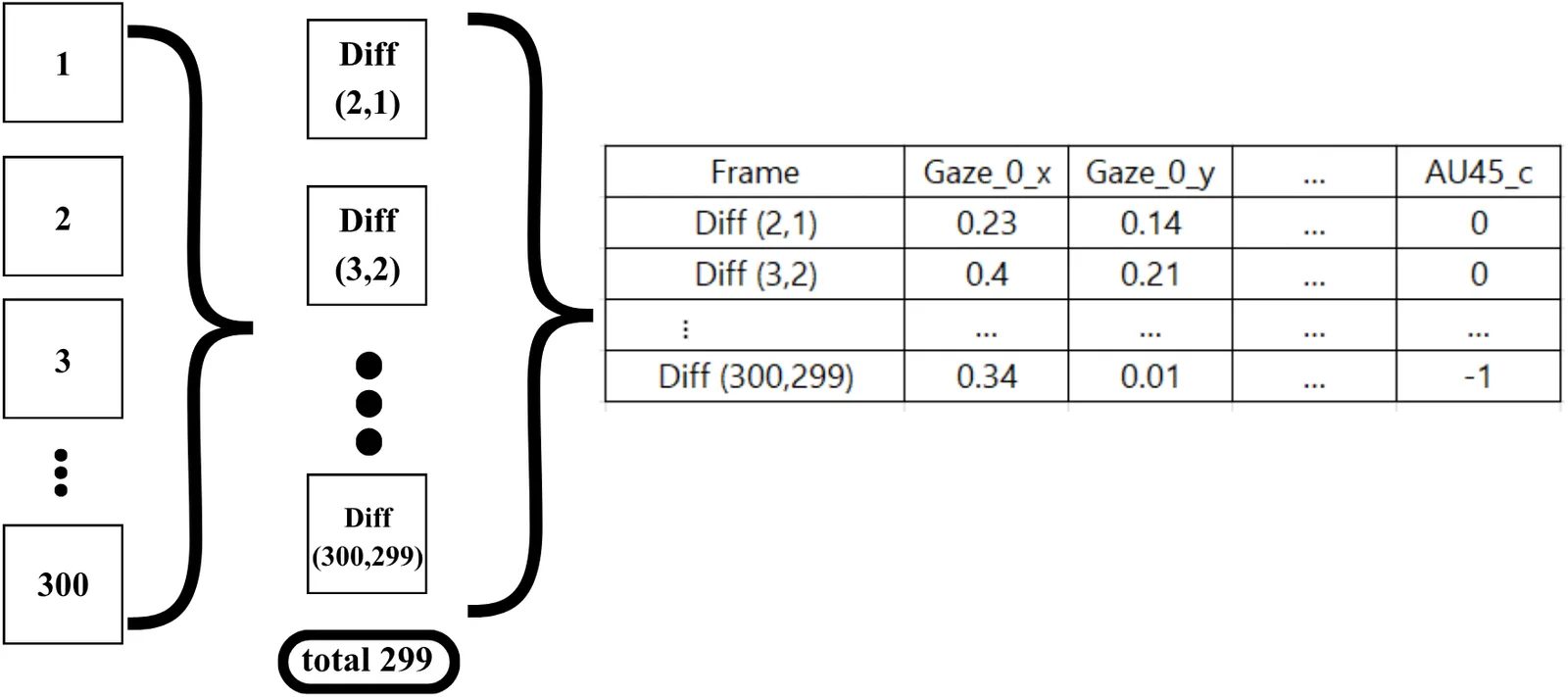
Frame differencing.

**Frame Differencing** Calculating differences between consecutive frames, producing 299 rows of 709 columns, then flattened to one row per participant (211,991 columns), highlighting subtle facial motion indicative of PD following Fig 7. The column counts reported above for each processing method correspond to the 10-second Original scenario (300 frames); for the 5-second Smile and Non-smile scenarios (150 frames), column counts scale accordingly (e.g., Sequential Frames: 106,350; Frame Skipping: 53,175; 3-Frame Averaging: 35,450; Frame Differencing: 105,641), while Frame Averaging remains a single 709-column vector regardless of duration. As the headline results reported in this study are based on the Smile scenario, these reduced dimensionalities describe the models actually evaluated.

All methods were evaluated to determine the strategy that provided the best results for classification and regression using accuracy, precision, recall, and RMSE.

### PCA dimensionality reduction

Principal Components Analysis or PCA was applied to reduce dimensionality and improve processing efficiency [24]. Three settings were tested:

**No PCA** Using all features without reduction.
**PCA 95% variance** Retaining components explaining 95% of variance (11–33 columns depending on processing method).
**PCA 98% variance** Retaining components explaining 98% of variance (20–49 columns depending on processing method).

These settings allowed comparison of model performance across compression levels and facilitated feature extraction for ML input.

### Modeling and evaluation

Three ML models were compared for PD classification and regression:

**Support Vector Machine (SVM)** Suitable for high-dimensional data, using an RBF kernel for nonlinear classification [25].

**Random Forest (RF)** An ensemble of decision trees that is robust to overfitting, captures nonlinear feature relationships, and provides feature importance estimates [26].

**XGBoost** A regularized gradient boosting framework with parallel processing and high efficiency for structured data [27].

To ensure reproducibility and maintain a consistent evaluation framework, all models were trained using the default hyperparameters of the Python packages: Scikit-learn version 1.6.1 [30] for the Random Forest and the Support Vector Machine models; and XGBoost version 3.0.3 [27] for the XGBoost model. This setting allows a comparison across models that is not confounded by unequal tuning effort. All stochastic components (bootstrap sampling, subsampling, and the cross-validation split) were fixed with a single random seed (random_state = 42). It should be noted that untuned defaults may understate the achievable performance of each model, particularly the Support Vector Machine, whose behaviour is sensitive to *C* and γ.

Models were trained and tested under various PCA configurations and video processing conditions. Classification performance was assessed using accuracy, precision, recall, and F1-score, whereas regression performance for Hoehn & Yahr stage and MDS-UPDRS score estimations were evaluated using RMSE and MAPE. Because healthy control participants were assigned a Hoehn & Yahr stage and MDS-UPDRS Part III score of zero by clinical definition, MAPE which is undefined when the true value is zero was computed using only the PD participants (n = 20), while RMSE was computed using all 40 participants.

Model performance was evaluated using 5-fold cross-validation [28]. The dataset was split into five equally sized folds in a patient-wise manner, ensuring that all samples from a single participant appeared exclusively in either the training or testing fold. Each fold served once as the test set, while the remaining four folds were used for training (approximately 80% training and 20% testing). This strategy reduces bias, prevents subject leakage, and provides a balanced and reliable model evaluation.

## Experiments and results

In this section, the experimental results and data analyses are presented in three parts: Descriptive Statistics, Experimental Results Analysis, and Explainable AI.

### Descriptive statistics

A total of 40 participants were included in this study, divided into two groups: 20 PD patients and 20 healthy controls. These groups were used for comparison to develop and evaluate ML models for automatic facial-based PD classification.

#### Demographics of participants.

Table 1 presents the demographic characteristics of both groups, including mean ± standard deviation of age, gender distribution, and clinical severity measures. The mean ± standard deviation of age of the PD group was 67.95 ± 9.57 years, while the healthy control group had a mean age of 70.15 ± 11.56 years. Age did not differ significantly between groups (Welch’s *t*-test, *p* = 0.52), helping to minimize potential age-related confounding in the dataset used for model training. Regarding gender distribution, the PD group comprised 11 males and 9 females, whereas the healthy con*t*rol group included 5 males and 15 females. The groups therefore differ in sex composition by 30 percentage points. Because sex may influence facial expressiveness and motor-related features, this imbalance is a potential confounder; it was not adjusted for in the models and is revisited in the Limitations. For the PD group, additional clinical measures were collected to quantify disease severity. The mean ± standard deviation of Levodopa Equivalent Daily Dose (LEDD) in the patients is 680.30 ± 470.90 mg/day; and the mean ± standard deviation motor examination scores (MDS-UPDRS Part III) is 29 ± 24.54 points, with the cohort spanning Hoehn & Yahr Stage 1 to Stage 5, indicating a wide range of motor symptom severity.

**Table 1:** Demographic characteristics of study participants.

Group	Age (years)	Male	Female	LEDD (mg/day)	MDS-UPDRS Part III
PD	67.95 ± 9.57	11	9	680.30 ± 470.90	29 ± 24.54
Control	70.15 ± 11.56	5	15	–	–

Age was compared using Welch’s *t*-test (*p* = 0.52); sex distribution was compared using Fisher’s exact test (*p* = 0.11).

#### Duration of Parkinson’s disease among Parkinson’s participants.

Table 2 summarizes the descriptive statistics related to the duration of Parkinson’s disease among participants diagnosed with PD. The mean ± standard deviation age at the onset of motor symptoms was 61.80 ± 16.96 years, while the mean ± standard deviation age at clinical diagnosis was 64.65 ± 12.76 years. This gap indicates the period during which patients first experienced symptoms before entering formal clinical evaluation.

**Table 2:** Duration and key clinical timelines of Parkinson’s disease among PD participants (years).

Group	Age at Onset of Motor Symptoms	Age at Clinical Diagnosis	Duration from Onset to Diagnosis
PD	61.80 ± 16.96	64.65 ± 12.76	2.75 ± 2.71

Additionally, the duration from the initial onset of motor symptoms to the time of diagnosis mean ± standard deviation is 2.75 ± 2.71 years. This duration was independently reported for each participant based on clinical or patient-recalled history, rather than derived by subtracting the mean onset age from the mean diagnosis age; the individual-level onset-to-diagnosis intervals therefore need not equal the difference between the two column means shown in Table 2. This delay suggests that most patients did not receive an immediate diagnosis following the emergence of symptoms, which may relate to disease awareness, access to medical services, or the subtle nature of early motor manifestations.

#### the Modified Hoehn & Yahr Scale of Parkinson’s disease among Parkinson’s participants.

Furthermore, the Modified Hoehn & Yahr Scale was used to assess PD severity. Unlike the original five-stage scale described in the Introduction, the Modified scale includes intermediate half-stages, yielding seven possible levels for PD participants (Stage 1, 1.5, 2, 2.5, 3, 4, and 5), with higher stages indicating greater disease severity; healthy controls were assigned a value of 0, as noted in the Participants and Data Collection section, giving the regression target eight distinct values overall. From the classification results, patients were distributed across stages as follows:

Table 3 shows that most patients were in Stage 3, representing the mid-stage of disease progression, where noticeable motor impairments begin but patients can still move independently. Additionally, some patients were in higher stages (Stage 4 and Stage 5), indicating severe symptoms and dependence on others for daily activities. Having data across a wide range of disease stages enhances the model’s ability to accurately differentiate between PD patients and healthy controls.

**Table 3:** Distribution of Parkinson’s patients according to disease severity (Modified Hoehn & Yahr Scale).

Group	Stage 1	Stage 1.5	Stage 2	Stage 2.5	Stage 3	Stage 4	Stage 5
PD	3	1	3	2	7	2	2

Presenting these descriptive statistics not only provides an overview of the sample population characteristics but also allows an initial assessment of whether the data are balanced and suitable for training ML models for facial-based PD classification, which is the primary objective of this study.

### Experimental results analysis

In each predictive model, experiments were conducted to determine which data handling and video processing methods provide the highest performance. The effect of facial expression scenarios on model performance is described in the Facial Action Scenarios section.

The performance of five video processing methods, described in the Feature Engineering via Data Processing Methods section, was also compared. Each dataset generated from these video processing methods was tested under different PCA dimensionality reduction conditions: No PCA, PCA explaining 95% of the variance, and PCA explaining 98% of the variance. Different settings were compared through the accuracy, precision, recall, and F1-score for classification and the RMSE and MAPE for regression.

#### Classification of Parkinson’s disease.

##### A: Video processing methods.

The results are presented in Table 4.The performance across the five video processing methods showed descriptive differences in how temporal structure and PCA-based dimensionality reduction influenced classification outcomes. For the Sequential Frames method, performance without PCA was stable, with accuracy = 0.71, precision = 0.71, recall = 0.69, and F1-score = 0.68 (all values in XGBoost). When PCA retained 95% variance, results remained comparable, achieving accuracy = 0.71, precision = 0.72, recall = 0.72, and F1-score = 0.71 (all values in Random Forest). However, when PCA retained 98% variance, performance decreased noticeably, with accuracy = 0.66, precision = 0.71, recall = 0.58, and F1-score = 0.64 (all values in XGBoost), suggesting that preserving excessive variance may introduce redundant temporal fluctuations that reduce model discriminability.

**Table 4:** Classification performance across video pre-processing methods, with and without PCA dimensionality reduction. Values are the best result across all four Facial Action Scenarios, and all metrics are means over the 5 cross-validation folds (the reported F1-score is not recomputed from mean precision and recall).

Video Method	No PCA				PCA retaining 95%				PCA retaining 98%			
	Acc.	Pre.	Re.	F1	Acc.	Pre.	Re.	F1	Acc.	Pre.	Re.	F1
Sequential Frames	0.71^*b*^	0.71	0.69	0.68	0.71^*a*^	0.72	0.72	0.71	0.66^*b*^	0.71	0.58	0.64
Frame Skipping	0.73^*b*^	0.72	0.75	0.73	**0.77** ^***b***^	**0.75**	**0.86**	**0.79**	0.75^*a*^	0.72	0.82	0.77
Frame Averaging	0.73^*b*^	0.73	0.68	0.61	0.71^*c*^	0.76	0.71	0.63	0.68^*c*^	0.66	0.66	0.64
3-Frame Averaging	0.69^*b*^	0.69	0.66	0.66	0.70^*c*^	0.63	1.00	0.71	0.69^*c*^	0.66	0.66	0.66
Frame Differencing	0.68^*a*^	0.68	0.92	0.64	0.57^*a*^	0.56	0.62	0.59	0.56^*a*^	0.58	0.48	0.50

^*a*^ Random Forest, ^*b*^ XGBoost, ^*c*^ Support Vector Machine.

The Frame Skipping method exhibited the highest observed overall performance. Without PCA, the method achieved accuracy = 0.73, precision = 0.72, recall = 0.75, and F1-score = 0.73 (all values in XGBoost). When PCA retaining 95% variance was applied, this method reached its best performance, with accuracy = 0.77, precision = 0.75, recall = 0.86, and F1-score = 0.79 (all values in XGBoost), representing the highest scores across all configurations. Even with PCA retaining 98% variance, performance remained strong, yielding accuracy = 0.75, precision = 0.72, recall = 0.82, and F1-score = 0.77 (all values in Random Forest). These results indicate that frame skipping helps discard redundant facial motion while maintaining essential temporal transitions for Parkinson’s detection.

The Frame Averaging method produced moderate results across conditions. Without PCA, performance included accuracy = 0.73, precision = 0.73, recall = 0.68, and F1-score = 0.61 (all values in XGBoost). With PCA retaining 95% variance, accuracy = 0.71, precision = 0.76, recall = 0.71, and F1-score = 0.63 were obtained with the Support Vector Machine. However, with PCA retaining 98%, performance declined to accuracy = 0.68 (SVM), with reduced precision and recall, indicating that averaging all frames removes temporal variations important for distinguishing Parkinson’s facial patterns.

The 3-Frame Averaging method showed similar limitations. Without PCA, the method produced accuracy = 0.69, precision = 0.69, recall = 0.66, and F1-score = 0.66 (all values in XGBoost). When PCA retained 95% variance, the method exhibited a very high recall of 1.00, but a low precision of 0.63 (all values in SVM), indicating a tendency to over-classify individuals as having Parkinson’s disease. With PCA retaining 98% variance, performance remained modest, with accuracy = 0.69, precision = 0.66, recall = 0.66, and F1-score = 0.66 (all values in SVM).

The Frame Differencing method showed high sensitivity without PCA, achieving recall = 0.92, although overall performance was moderate (accuracy = 0.68, precision = 0.68, F1-score = 0.64; all from Random Forest). Once PCA was applied, performance dropped. With PCA retaining 95% variance, results were accuracy = 0.57, precision = 0.56, recall = 0.62, and F1-score = 0.59 (all values in Random Forest). When PCA retained 98% variance, the decline was more severe, with accuracy = 0.56, precision = 0.58, recall = 0.48, and F1-score = 0.50 (all values in Random Forest). These findings suggest that PCA removed subtle frame-to-frame motion differences essential for accurate classification.

##### B: Facial action scenarios.

The results are presented in Table 5. The classification results across the four facial action scenarios, as presented in Table 5, illustrate how different types of facial movements affect the model’s ability to detect Parkinson’s disease. For the Original scenario, which mixes neutral and smiling expressions within the same video, the Support Vector Machine achieved moderate performance with accuracy = 0.68, precision = 0.71, recall = 0.68, and F1-score = 0.64. Although combining both expressions offers a broader representation of facial behavior, the mixed temporal and structural patterns introduce substantial variability that may obscure the disease-specific facial motion cues. This reduces the model’s ability to extract stable and discriminative features.

**Table 5:** Classification performance of Facial Action Scenarios. Each row reports the best configuration for that scenario, so the processing method and PCA setting differ across rows; metrics are means over the 5 cross-validation folds (the reported F1-score is not recomputed from mean precision and recall).

Facial Action Scenarios	Accuracy	Precision	Recall	F1-score
Original ^*c*^	0.68	0.71	0.68	0.64
Non-smile ^*a*^	0.71	0.73	0.67	0.69
Smile ^*b*^	**0.77**	**0.75**	**0.86**	**0.79**
Combined ^*c*^	0.66	0.68	0.68	0.65

a Random Forest, ^*b*^ XGBoost, ^*c*^ Support Vector Machine.

The Non-smile scenario showed slightly improved performance, with accuracy = 0.71, precision = 0.73, recall = 0.67, and F1-score = 0.69, using the Random Forest classifier. Neutral facial expressions provide stable structural information, especially around the upper face, allowing the model to capture consistent geometric patterns. However, the lack of strong dynamic motion limits the visibility of hypomimia-related impairments. This leads to higher precision but lower recall, indicating that individuals with mild or subtle symptoms may be under-detected.

The Smile scenario demonstrated the highest classification performance across all metrics. Using XGBoost, the model achieved accuracy = 0.77, precision = 0.75, recall = 0.86, and F1-score = 0.79. Smiling engages multiple facial muscle groups, generating dynamic and expressive motion patterns that better reveal motor impairments characteristic of Parkinson’s disease. These expressive actions amplify the contrast between patients and healthy controls, enabling the model to capture fine-grained deficits more effectively. The notably high recall reflects strong sensitivity, suggesting that smiling expressions provide the richest diagnostic information for automatic classification.

Finally, the Combined scenario, despite containing the largest number of videos (400 samples), yielded lower performance with the Support Vector Machine (accuracy = 0.66, precision = 0.68, recall = 0.68, F1-score = 0.65). Although combining all scenarios increases dataset size, it also introduces heterogeneous and sometimes conflicting feature distributions. The mixture of static and dynamic expressions may dilute scenario-specific cues and create redundant or competing patterns, ultimately reducing classification stability and overall accuracy.

Collectively, these results suggest that facial expressions involving active movement, particularly smiling, contain the most diagnostically relevant features for distinguishing Parkinson’s disease from healthy controls. In contrast, mixing different expression types or relying solely on neutral expressions decreases classification performance due to increased variability or insufficient dynamic information.

#### Parkinson’s severity Hoehn and Yahr scale.

##### A: Video frame methods.

The results are presented in Table 6. The evaluation of video processing methods for the Hoehn & Yahr severity estimation model revealed clear differences in how each technique preserves clinically meaningful facial-motion information. For the Sequential Frames method, the Random Forest produced an RMSE of 1.33 and a MAPE of 39.32% when PCA was not applied. Under PCA retaining 95% and 98% of the explained variance, the XGBoost model yielded higher errors (RMSE = 1.53, MAPE = 40.56%; RMSE = 1.54, MAPE = 41.19%), indicating that dimensionality reduction may remove subtle yet important temporal cues necessary for capturing continuous facial-motor patterns associated with Parkinson’s disease severity.

**Table 6:** RMSE and MAPE of Hoehn & Yahr regression across video processing methods. Values are the best result across all four Facial Action Scenarios.

Video Method	No PCA		PCA retaining 95%		PCA retaining 98%	
	RMSE	MAPE	RMSE	MAPE	RMSE	MAPE
Sequential Frames	1.33^*a*^	39.32%	1.53^*b*^	40.56%	1.54^*b*^	41.19%
Frame Skipping	**1.30** ^*a*^	**38.36%**	1.47^*c*^	42.32%	1.49^*c*^	43.94%
Frame Averaging	1.31^*a*^	35.59%	1.54^*b*^	40.21%	1.46^*a*^	42.10%
3-Frame Averaging	1.32^*a*^	37.89%	1.52^*a*^	42.35%	1.53^*a*^	42.77%
Frame Differencing	1.47^*a*^	46.95%	1.69^*a*^	54.10%	1.60^*a*^	50.82%

^*a*^ Random Forest, ^*b*^ XGBoost, ^*c*^ Support Vector Machine.

RMSE computed on all 40 participants; MAPE computed on PD participants only (n = 20).

For the Frame Skipping method, the lowest RMSE among all methods was achieved by the Random Forest (RMSE = 1.30, MAPE = 38.36%) without PCA, although a lower MAPE (35.59%) was obtained with Frame Averaging without PCA. However, when PCA retaining 95% and 98% was applied, the Support Vector Machine model showed increased errors (RMSE = 1.47, MAPE = 42.32%; RMSE = 1.49, MAPE = 43.94%). These results suggest that while frame skipping effectively reduces redundancy, additional dimensionality reduction may discard clinically relevant facial-motion features.

For the Frame Averaging method, the Random Forest produced RMSE = 1.31 and MAPE = 35.59% when PCA was not used. After applying PCA, XGBoost yielded higher errors (RMSE = 1.54, MAPE = 40.21%), while Random Forest also showed increased error at the 98% level (RMSE = 1.46, MAPE = 42.10%). This indicates that averaging across all frames may limit the ability to capture subtle fluctuations in facial expressiveness, and PCA may further weaken discriminative facial-motion information.

For the 3-Frame Averaging method, without PCA, the Random Forest achieved RMSE = 1.32 and MAPE = 37.89%. Under PCA at 95% and 98%, Random Forest continued to produce higher errors (RMSE = 1.52, MAPE = 42.35%; RMSE = 1.53, MAPE = 42.77%). This suggests that while frame smoothing reduces noise, it may also attenuate clinically meaningful fine-grained motion patterns, and PCA amplifies this loss.

Lastly, the Frame Differencing method produced the highest prediction errors overall. Using the Random Forest without PCA resulted in RMSE = 1.47 and MAPE = 46.95%. When PCA retaining 95% and 98% was applied, even higher errors were produced by the Random Forest (RMSE = 1.69, MAPE = 54.10%; RMSE = 1.60, MAPE = 50.82%). This reflects the limitation of relying solely on rapid frame-to-frame changes, which do not preserve the gradual progression of facial-motor patterns characteristic of Parkinson’s disease.

Overall, the results demonstrate that methods preserving temporal structure, particularly frame Skipping and mean-based transformations, provide the most reliable predictive performance across machine learning models. Conversely, PCA generally increases both RMSE and MAPE, suggesting that the Hoehn & Yahr estimation task depends on high-dimensional facial-motion information that may be overly compressed by dimensionality reduction. As a reference baseline, a naive model that always predicts the sample mean Hoehn & Yahr stage would achieve an RMSE of approximately 1.59 (the population standard deviation of the target across all 40 participants); the best-performing model (RMSE = 1.30) therefore corresponds to an approximate *R*^2^ of 0.33.

##### B: Facial action scenarios.

The results are presented in Table 7.

**Table 7:** RMSE and MAPE of Hoehn & Yahr regression across Facial Action Scenarios. Each row reports the best configuration for that scenario, so the processing method and PCA setting differ across rows.

Facial Action Scenarios	Root Mean Squared Error	Mean Absolute Percentage Error
Original ^*a*^	1.31	35.59%
Non-smile ^*a*^	1.56	45.39%
Smile ^*a*^	**1.30**	**38.36%**
Combined ^*b*^	1.54	41.69%

^*a*^ Random Forest, ^*b*^ XGBoost, ^*c*^ Support Vector Machine.

RMSE computed on all 40 participants; MAPE computed on PD participants only (n = 20).

The analysis of RMSE and MAPE across different facial action scenarios in the Hoehn & Yahr severity model reveals consistent patterns regarding the predictive value of facial expressions. The Original Video scenario, evaluated using the Random Forest, achieved an RMSE of 1.31 and MAPE of 35.59%, indicating relatively good predictive stability. Using the full 10-second segment allows the model to capture both neutral and expressive behaviors, providing a balanced representation of facial-motor activity.

The Non-smile scenario, also based on Random Forest, produced the highest error values (RMSE = 1.56; MAPE = 45.39%). The substantially higher MAPE suggests that predictions deviate more proportionally from true Hoehn & Yahr scores when only neutral facial segments are used. The lack of expressive movement limits the availability of dynamic motor cues, making it difficult for the model to detect hypomimia-related severity levels.

In contrast, the Smile scenario, again modeled using Random Forest, achieved the lowest RMSE of 1.30 and a moderate MAPE of 38.36%. Although its MAPE is slightly higher than that of the Original scenario, the RMSE indicates superior absolute prediction accuracy. Smiling activates multiple muscle groups and produces stronger dynamic contrasts between Parkinson’s patients and controls, offering more informative features for the model to learn from.

The Combined scenario, analyzed with the XGBoost model, yielded RMSE = 1.54 and MAPE = 41.69%. While its MAPE is lower than that of the Non-smile scenario, both error metrics are worse compared to using smile alone. The mixing of two different facial-expression dynamics likely introduces feature heterogeneity, reducing the model’s ability to extract consistent severity-related patterns.

Overall, the integration of both RMSE and MAPE metrics confirms that facial expressions involving active movement, particularly smiling, provide the most reliable cues for Hoehn & Yahr severity estimation. Models trained only on neutral segments or mixed heterogeneous segments tend to perform worse due to insufficient or inconsistent motion information.

To contextualize the clinical significance of these error metrics, the distribution of prediction errors relative to adjacent Hoehn & Yahr stages was analyzed. For the best-performing model (Frame Skipping + Smile + No PCA), 72.50% of predictions fell within ±1 stage of the true Hoehn & Yahr value, and 92.50% fell within ±2 stages.Given that clinical assessments by different neurologists can vary due to inter-rater variability in Hoehn & Yahr staging [31], these results suggest that the facial video-based predictions achieve clinically acceptable agreement. The MAPE of 38.36% should be interpreted in the context of the ordinal nature of the Hoehn & Yahr scale, where percentage errors are amplified at lower severity stages (*e.g.*, an estimation of Stage 2 versus true Stage 1 yields 100% error despite being clinically adjacent).

#### Parkinson’s motor scores (MDS-UPDRS Part III).

##### A: Video processing methods.

The results are presented in Table 8. The evaluation of RMSE and MAPE across different video processing methods for predicting MDS-UPDRS Part III scores, as presented in Table 8, indicates clear differences in model performance depending on how temporal facial information is represented.

**Table 8:** RMSE and MAPE for MDS-UPDRS Part III across video processing methods. Values are the best result across all four Facial Action Scenarios.

Video Method	No PCA		PCA retaining 95%		PCA retaining 98%	
	RMSE	MAPE	RMSE	MAPE	RMSE	MAPE
Sequential Frames	20.85^*a*^	51.97%	20.13^*a*^	46.49%	20.61^*a*^	47.88%
Frame Skipping	20.63^*a*^	52.53%	20.47^*c*^	47.51%	20.62^*c*^	47.74%
Frame Averaging	20.83^*a*^	51.49%	19.78^*a*^	44.62%	22.06^*c*^	49.86%
3-Frame Averaging	20.23^*a*^	51.33%	**19.34** ^*b*^	**45.33%**	19.37^*b*^	45.87%
Frame Differencing	21.94^*a*^	57.27%	23.36^*a*^	61.45%	22.54^*a*^	60.32%

^*a*^ Random Forest, ^*b*^ XGBoost, ^*c*^ Support Vector Machine.

RMSE computed on all 40 participants; MAPE computed on PD participants only (n = 20).

The Sequential Frames method, which preserves every frame, produced RMSE values between 20.13 and 20.85 and corresponding MAPE values ranging from 46.49% to 51.97%, with all performance values obtained using Random Forest. Using PCA retaining 95% variance resulted in the lowest RMSE (20.13), indicating that moderate dimensionality reduction helps remove redundancy while retaining relevant temporal information.

The Frame Skipping method yielded RMSE values between 20.47 and 20.63 and MAPE between 47.51% and 52.53% with performance values using Random Forest in No PCA and Support Vector Machine in PCA retaining 95% and 98%. Although performance was comparable to sequential frames, this method provides computational advantages by reducing the number of processed frames without substantially degrading predictive accuracy.

The Frame Averaging method, which averages all frames into a single representation, resulted in RMSE values between 19.78 and 22.06 and MAPE between 44.62% and 51.49%. Performance values were obtained using Random Forest without PCA and with PCA retaining 95%, and using Support Vector Machine with PCA retaining 98%. The lowest RMSE and MAPE in this method were achieved under PCA (95%), suggesting that global temporal averaging combined with dimensionality reduction helps stabilize feature representation.

The 3-Frame Averaging method demonstrated the best overall performance, with RMSE values of 19.34–20.23 and MAPE of 45.33–51.33% with performance values using Random Forest in No PCA and XGBoost in PCA retaining 95% and 98%. The lowest RMSE across all methods (19.34) was achieved using XGBoost with PCA retaining 95% variance, while the lowest MAPE (44.62%) was obtained by Frame Averaging with PCA retaining 95% variance. This approach preserves important short-term temporal patterns while smoothing noise, making it particularly suitable for capturing facial-motor characteristics associated with Parkinson’s disease.

Finally, the Frame Differencing method consistently produced the highest errors, with RMSE values between 21.94 and 23.36 and MAPE between 57.27% and 61.45%, with all performance values obtained using Random Forest. Computing differences amplifies small variations across frames, introducing instability and high-frequency noise that impair model learning.

In summary, the integration of RMSE and MAPE results shows that temporal averaging approaches, especially short interval averaging paired with PCA, offer the most reliable representation for predicting MDS-UPDRS Part III severity. By contrast, methods relying on raw or difference-based temporal information exhibit greater variability and reduced predictive accuracy. As a reference baseline, a naive model that always predicts the sample mean MDS-UPDRS Part III score would achieve an RMSE of approximately 22.3 (the population standard deviation of the target across all 40 participants); the best-performing model (RMSE = 19.34) therefore corresponds to an approximate *R*^2^ of 0.25.

##### B: Facial action scenarios.

The analysis of RMSE and MAPE across different facial action scenarios, as shown in Table 9, demonstrates that the type of facial expression substantially influences the predictive performance of the MDS-UPDRS Part III model. The Original scenario, evaluated using the Random Forest, produced an RMSE of 20.13 and a MAPE of 46.49%. Although the full 10-second video contains the largest amount of information, the mixture of both neutral and smiling expressions may introduce variability that limits predictive precision.

**Table 9:** RMSE and MAPE for MDS-UPDRS Part III across Facial Action Scenarios. Each row reports the best configuration for that scenario, so the processing method and PCA setting differ across rows.

Facial Action Scenarios	Root Mean Squared Error	Mean Absolute Percentage Error
Original ^*a*^	20.13	46.49%
Non-smile ^*a*^	21.94	57.27%
Smile ^*b*^	**19.34**	**45.33%**
Combined ^*c*^	20.47	47.51%

a Random Forest, ^*b*^ XGBoost, ^*c*^ Support Vector Machine.

RMSE computed on all 40 participants; MAPE computed on PD participants only (n = 20).

The Non-smile scenario, also modeled using Random Forest, resulted in the highest error values (RMSE = 21.94; MAPE = 57.27%). Since neutral facial segments contain minimal expressive movement, the model receives limited dynamic motor information. This restriction reduces its ability to distinguish hypomimia patterns associated with different severity levels of Parkinson’s disease.

In contrast, the Smile scenario, evaluated using the XGBoost model, achieved the best predictive performance, with the lowest RMSE of 19.34 and the lowest MAPE of 45.33%. Smiling activates multiple facial muscle groups and generates dynamic, high-contrast motion cues that allow the model to more effectively capture motor impairments. These expressive features provide strong discriminatory power for predicting MDS-UPDRS Part III scores.

The Combined scenario, analyzed using the SVM, yielded RMSE = 20.47 and MAPE = 47.51%. Despite integrating both neutral and smiling segments, the heterogeneity of expression dynamics introduces inconsistency in the feature space, resulting in reduced performance when compared with the smiling-only scenario.

Overall, the results indicate that dynamic and expressive facial movements, particularly smiling, provide the most informative cues for predicting MDS-UPDRS Part III severity. In contrast, neutral or mixed-expression segments offer less consistent motion information, leading to higher estimation errors.

The clinical interpretation of the RMSE (19.34 points) and MAPE (45.33%) requires consideration of the MDS-UPDRS Part III score range and clinically meaningful differences. The MDS-UPDRS Part III scale spans 0–132 points, and a change of 3.25 points is considered the Minimal Clinically Important Difference (MCID) [32]. The model’s RMSE of approximately 19 points corresponds to roughly six times the MCID, indicating room for improvement.

### Explainable AI

In this study, feature importance was evaluated using the best-performing model from each of the following tasks: Parkinson’s Disease Classification, Parkinson’s Disease Severity Estimation, and Motor Score Estimation (MDS-UPDRS Part III).

To overcome the challenges of PCA interpretation, two procedures were implemented:

- **Without PCA** – Feature importance was extracted directly from the trained models, allowing influential facial variables to be identified in their original form.

Feature importance was computed using each model’s built-in importance measure. For the Random Forest regression model (used for Hoehn & Yahr severity estimation), importance was calculated as the mean decrease in variance attributable to each feature across all trees in the ensemble [26]:


$$\begin{array}{c} Imp(X_{j})=\frac{1}{T}\sum_{t=1}^{T}\sum_{n\in N_{t}:v(s_{n})=j}\frac{N_{n}}{N}\Delta \sigma^{2}(s_{n},n) \end{array}$$ (1)

where *T* is the total number of trees, $N_{t}$ denotes the nodes of tree *t*, $v(s_{n})$ indicates the feature used *t*o split node *n*, $N_{n}/N$ is the proportion of samples reaching node *n*, and $\Delta \sigma^{2}(s_{n},n)$ is the decrease in variance resulting from the split.

For the XGBoost models (used for PD classification and MDS-UPDRS Part III motor score estimation), feature importance was computed using the gain-based metric, reflecting the average improvement in the objective function contributed by each feature across all boosting trees [27]:


$$\begin{array}{c} Imp(X_{j})=\sum_{t=1}^{T}\sum_{s\in S_{t}:v(s)=j}Gain(s) \end{array}$$ (2)

where the gain of a candidate split *s* is defined as:


$$\begin{array}{c} Gain=\frac{1}{2}\left[\frac{G_{L}^{2}}{H_{L}+\lambda }+\frac{G_{R}^{2}}{H_{R}+\lambda }-\frac{(G_{L}+G_{R}{)}^{2}}{H_{L}+H_{R}+\lambda }\right]-\gamma \end{array}$$ (3)

with $G_{L},G_{R}$ and $H_{L},H_{R}$ representing the sums of the first- and second-order gradients of the loss (or objective) function for the left and right child nodes, respectively, λ the L2 regularization term, and γ the minimum loss reduction required for a split.

- **With PCA** – The model was trained using principal components (PCs). After training, the PCs that strongly influenced the model were identified, and their loadings were mapped back to the original variables to determine which facial movements contributed most to those components.

Feature importance was computed through a three-step procedure. First, the original features were transformed into principal components:


$$\begin{array}{c} Z=XW \end{array}$$ (4)

where *X* denotes the matrix of original features, *W* the loading matrix obtained from PCA, and *Z* the resulting matrix of principal components. Second, the importance of each component $Z_{k}$ was computed using Equation (1) or Equation (2), depending on the underlying model, by treating each component as an input feature, and the component with the highest importance was identified:


$$\begin{array}{c} k^{*}=\text{arg}\max_{k}\;Imp(Z_{k}) \end{array}$$ (5)

Third, the loadings of the selected component $Z_{k^{*}}$ were used directly to recover the contribution of each original facial feature:


$$\begin{array}{c} Imp_{back}(X_{j})=|W_{j,k^{*}}| \end{array}$$ (6)

where $W_{j,k^{*}}$ denotes the loading of original feature $X_{j}$ on the selected principal component $k^{*}$. This approach identifies the original facial features that most strongly underlie the most influential component, thereby preserving interpretability despite dimensionality reduction.

The PCA back-mapping process was designed to preserve interpretability even after dimensionality reduction. Although each PC is a transformed combination of many original features, the loadings indicate how strongly each original variable contributes to each component. By linking a highly influential PC back to the variables with the highest loadings, it is possible to determine which facial landmarks or motion patterns were responsible for the model’s decision. This approach allows the use of PCA for performance improvements while still retaining a clear explanation of the underlying facial features that drive model predictions.

The best-performing model from each of the three prediction tasks was analyzed to determine feature importance. All data were used as training data to identify which features contributed most to the models. The threshold for selecting influential features was determined using a visual elbow-point method: feature importance values (or absolute component loadings, for PCA-derived features) were sorted in ascending order as $v_{1}\leq v_{2}\leq ...\leq v_{M}$. The elbow point was identified as the index of maximum gap:


$$\begin{array}{c} i^{*}=\text{arg}\max_{i}\;\Delta_{i} \end{array}$$ (7)

corresponding to the point of steepest increase in the sorted importance sequence, which marks the transition from a low, near-uniform importance region to a region of rapidly increasing importance. This point was confirmed visually against the plotted importance curve to ensure consistency with the observed distribution. Features above this elbow point were retained as the top influential features for each predictive task, yielding the following counts:

1. Parkinson’s disease Classification Model: 404 features, selected via back-mapped PCA loadings (Equation 6), corresponding to the Frame Skipping configuration with PCA retaining 95% variance;
2. Parkinson’s disease Severity Estimation Model: 60 features, selected directly from the untransformed feature space (Equation 1), corresponding to the Frame Skipping configuration without PCA;
3. Motor Score Estimation Model (MDS-UPDRS Part III): 138 features, selected via back-mapped PCA loadings (Equation 6), corresponding to the 3-Frame Averaging configuration with PCA retaining 95% variance.

In each case, the threshold corresponds to the elbow point $i^{*}$ identified as the index of maximum gap $\Delta_{i}$ in the sorted importance sequence, as defined in Equation (7).

After feature importance was obtained for both PCA and non-PCA conditions, the most influential features were visualized by plotting representative frames along with their facial landmarks. These visual and numerical analyses provide insight into which facial characteristics are most critical for Parkinson’s disease classification, severity estimation, and motor score estimation, supporting model refinement and guiding future research.

#### Parkinson’s Disease prediction model.

**Fig 8:**
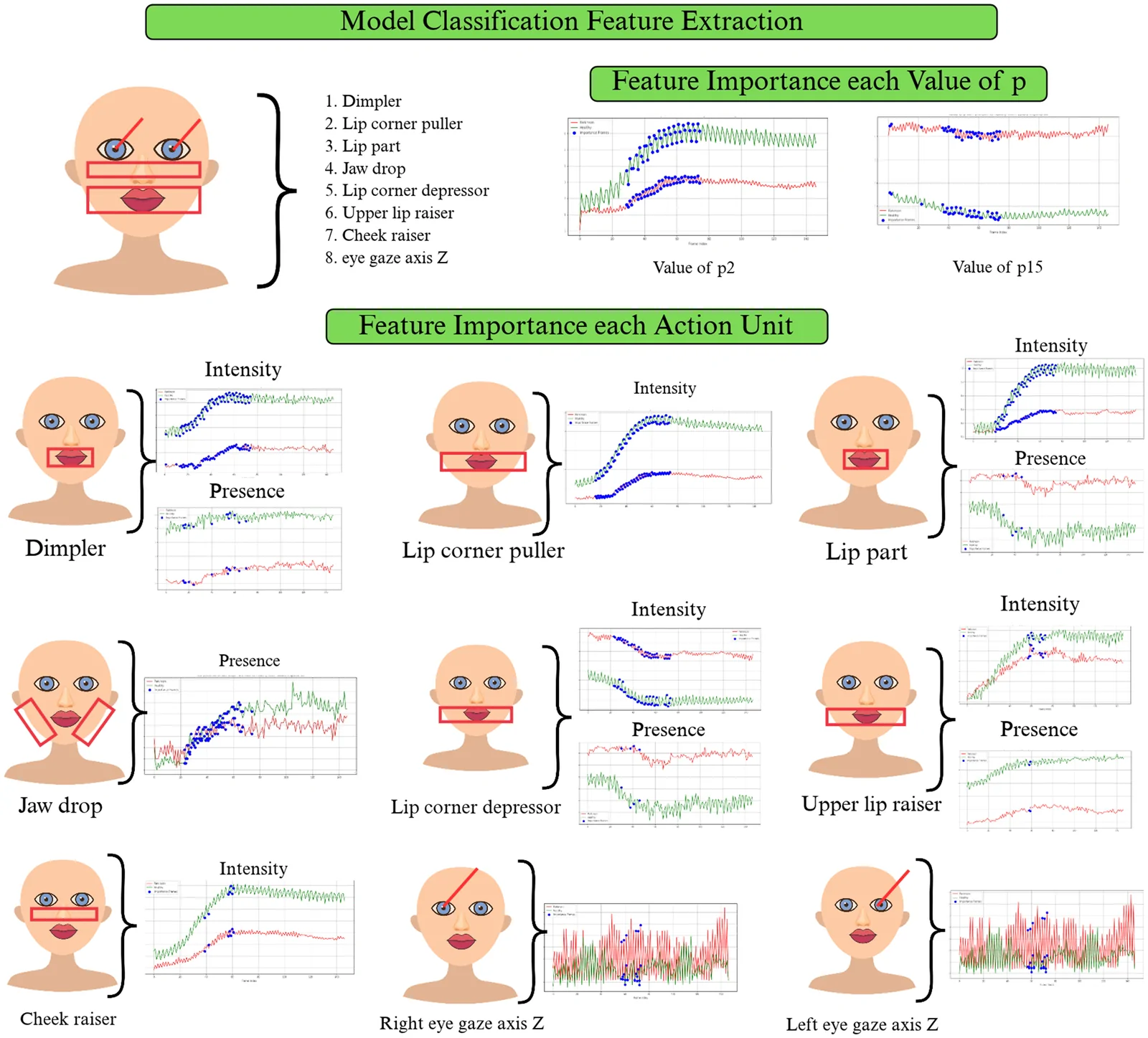
Overview of feature extraction for Parkinson’s disease prediction model.

For the Parkinson’s disease prediction model illustrated in Fig 8, the analysis of facial dynamics revealed that distinct groups of features contributed to the discrimination between patients and healthy controls. These features can be understood in terms of three primary components: facial Action Units (AUs), geometric landmark movements, and head gaze-related characteristics. Together, they provide a comprehensive representation of both expressive and subtle motor functions that are clinically relevant to Parkinson’s disease.

In terms of facial action units, movements around the lips and jaw demonstrated the strongest discriminative power. The intensity of the *dimpler* appeared consistently across early to mid ranges of the video, showing that lip corner tension and tightening play a prominent role in differentiating patient facial behavior. Similarly, the *lip corner puller* contributed substantially, particularly during the mid-to-late frames, where the lifting of the mouth corners becomes more pronounced. The *lip part* and *jaw drop* features further highlighted the importance of mouth opening and lower lip flexibility, both of which tend to be reduced in individuals with Parkinson’s disease due to hypomimia and bradykinesia. Additional AUs such as the *lip corner depressor*, *upper lip raiser*, and *cheek raiser* provided nuanced indicators of weakened or delayed expressive muscle activation, especially in the latter segments of the video where voluntary expressions typically occur. Even small-scale micro-expressions, including brief upper lip raising observed near the end of the sequence, contributed meaningful cues to the model.

The geometric information encoded by facial landmarks further enriched the interpretation. Landmark trajectories around the mouth, cheeks, and jawline captured subtle shape deformations over time, offering a continuous representation of expressive behavior. These geometric variations were reinforced by latent shape parameters such as *p2* and *p15*, which summarized global facial configuration changes across the full video. These parameters provided evidence that Parkinson’s patients exhibit reduced variability and slower transitions in facial shape, reflecting characteristic deficits in spontaneous facial movement.

Head and gaze-related information also played a significant role, particularly in the second half of the video. Gaze vectors extracted from both eyes revealed reduced saccadic activity and limited directional shifts, which have been associated with motor control impairment in Parkinson’s disease. The inclusion of these gaze features allowed the model to capture changes in ocular behavior that are not directly tied to facial muscle activation but nevertheless reflect neurological motor dysfunction. Combined with head pose stability and movement patterns, these gaze-based cues contributed additional context that strengthened the model’s ability to distinguish between patient and control groups.

Taken together, the integration of AUs intensities, landmark-based facial geometry, and gaze-related features demonstrated that the most informative patterns arise from dynamic and multi-regional facial behavior. Movements of the lips, jaw, and cheeks, combined with characteristic gaze behavior and global shape deformation, formed a coherent set of temporal indicators that reliably supported the accurate prediction of Parkinson’s disease across different segments of the video.

#### Parkinson’s disease severity estimation model.

**Fig 9:**
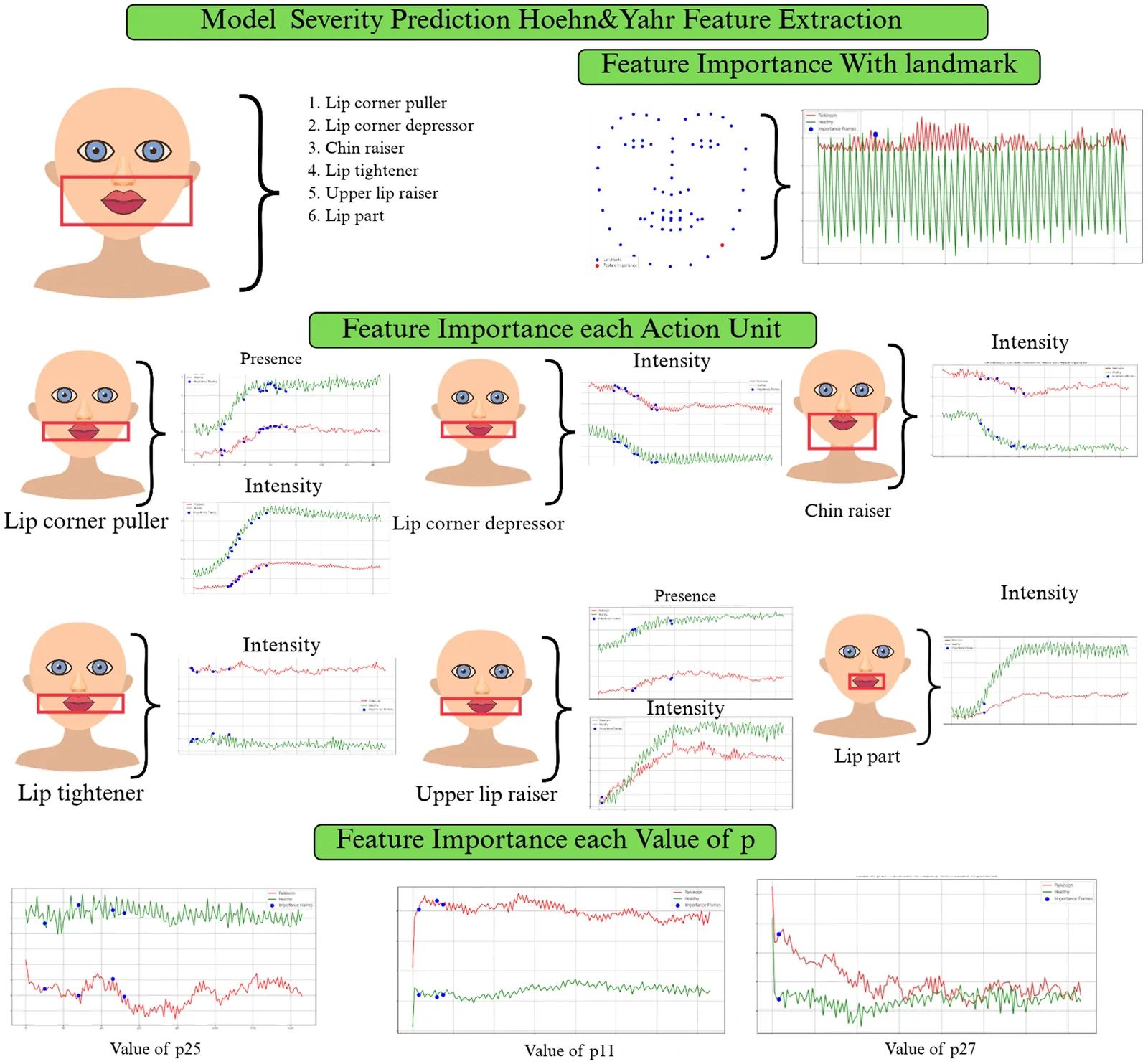
Overview of feature extraction for Parkinson’s disease severity prediction model of Hoehn and Yahr.

In the Parkinson’s disease severity estimation model based on Hoehn & Yahr staging, as illustrated in Fig 9, the analysis revealed that three major groups of facial information: Action Units (AUs), facial landmarks, and latent shape parameters play essential roles in distinguishing the disease severity across different temporal segments of the video.

For the *Action Units*, several perioral AUs emerged as highly informative. The presence of the *lip corner puller* across frames 20–80 indicated that the degree of mouth-corner lifting during mid-to-late portions of the video is strongly associated with severity levels. Complementing this, the intensity of the *lip corner depressor* in the same frame window reflected the extent of downward lip-corner movements, highlighting reduced voluntary expressiveness typically seen in more severe cases. Mid-video dynamics of the *lip corner puller intensity* further emphasized that not only the presence but also the strength of mouth-corner movement contributes meaningfully to severity discrimination. Additional cues were provided by the *chin raiser intensity* across frames 20–70, capturing lower-facial muscular tension consistent with bradykinesia-related expressivity reduction. Early facial rigidity was also reflected through the *lip tightener intensity* between frames 0–40. Upper-lip behavior contributed additional subtle distinctions: the presence and intensity of the *upper lip raiser* captured small vertical movements relevant to differentiating certain severity levels, while mid-video activation of the *upper lip part* added further detail regarding lip separation characteristics.

For the facial landmarks, the model identified a horizontal displacement in landmark 11 around frame 29 as a notable predictor. This slight left–right chin-region shift reflects micro-positional variations that differ systematically with disease severity. Such landmark-based positional information suggests that even minimal deviations in facial motion amplitude or symmetry can be meaningful indicators for Hoehn & Yahr classification.

For the latent shape parameters, represented by PCA-derived facial shape components, the analysis revealed complementary insights by capturing holistic geometric changes rather than isolated points or muscles. Components such as *p25*, detected across frames 0–60, and *p15* and *p11* in early frames, described broad shape deformations that develop during speech or voluntary movement. These components summarize patterns such as flattening, tightening, or drooping of facial regions that align with severity-related changes in overall expressivity. The contribution of *p27* at frame 5 further indicated that even early subtle shape deviations contribute predictive value prior to more explicit motion emerging later in the sequence.

Taken together, these findings demonstrate that the severity estimation model relies on a multilayered interaction between muscular activations captured through AUs, localized movements captured through facial landmarks, and global structural adjustments captured through latent shape parameters. The temporal distribution of these cues, from early-frame rigidity to mid- and late-frame movement strength, highlights the progressive nature of Parkinsonian facial expressivity and reinforces the interpretability of the model within an explainable AI framework.

#### Motor score estimation model (MDS-UPDRS Part III).

**Fig 10:**
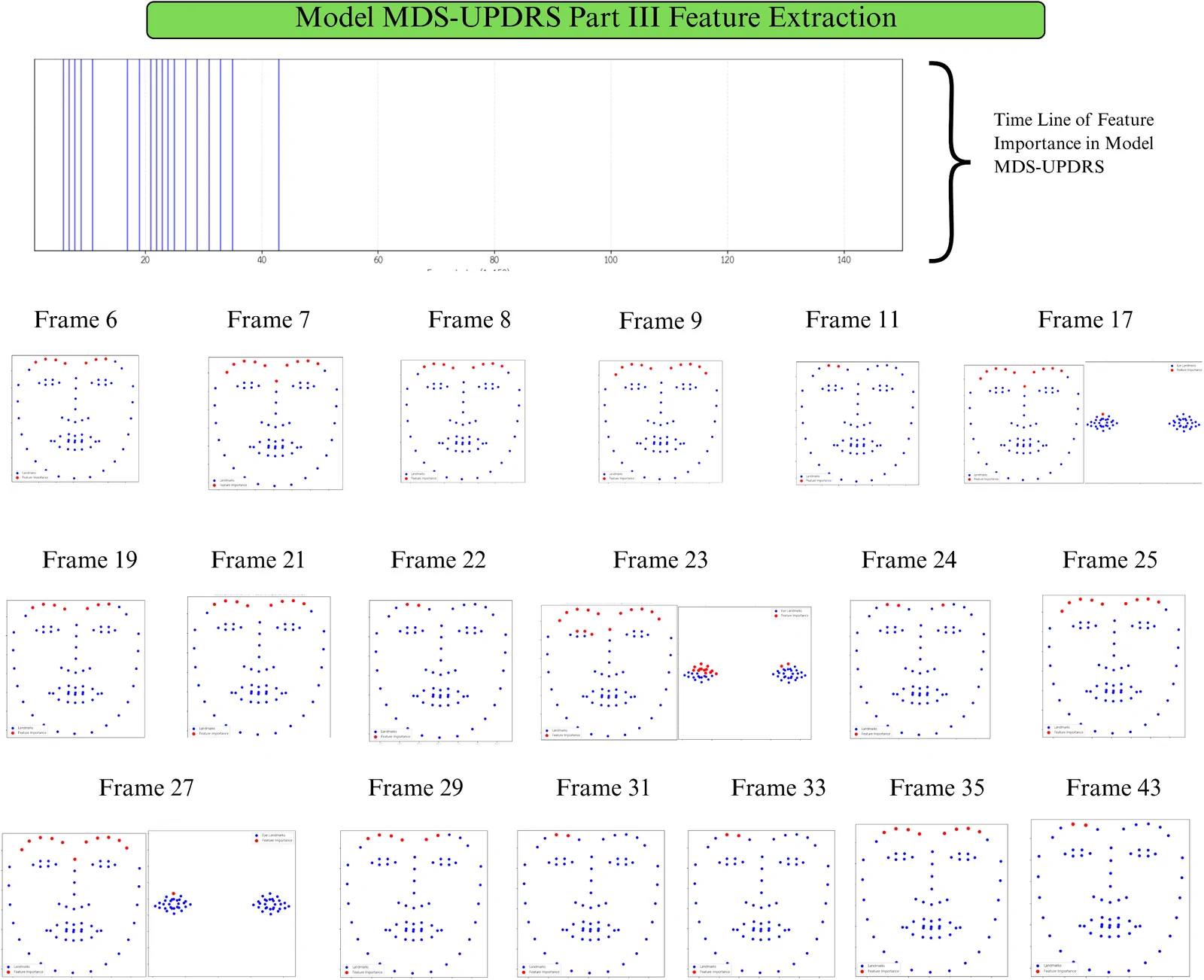
Overview of feature extraction for the MDS-UPDRS Part III model.

In the MDS-UPDRS Part III model, as illustrated in Fig 10, the predictive framework relies primarily on facial and ocular landmarks to estimate motor severity and characterize participants’ motion behavior. The analysis showed that temporal patterns of movement in the *eyebrows* and *eyes* provide the most discriminative information, with the majority of influential key points concentrated between frames 6 and 43. Within this range, the mid-video frames, particularly frames 17, 23, and 27, contained the highest density of informative points, indicating that expressive fluctuations during these moments carry strong predictive weight for motor symptom severity.

Early-frame activity, represented by 6–10 key points distributed across both eyebrows between frames 6–9, captured initial periocular muscle engagement. These early upward or downward micro-adjustments around the brow region reflect foundational motor behavior cues often associated with rigidity or bradykinesia. Additional early variation, such as the two key points identified on the right eyebrow at frame 11, highlighted subtle inter-individual differences in expressivity that the model leverages when separating mild from more pronounced motor impairments.

As the video progresses into the mid-section, the model identifies markedly stronger motion signals. Frame 17, for example, contains 11 key points on the eyebrows alongside a single key point from the right eye, reflecting coordinated brow eye interactions that frequently accompany intentional or reflexive facial adjustments. Frame 23 stands out as a particularly informative moment, with 29 total key points (14 from the eyebrows and 15 from the eyes). This dense concentration of motion features indicates a segment where expressive intensity peaks, offering the model a rich representation of ocular and periocular dynamics, such as blinking amplitude, eyebrow lifting, or brow compression behaviors known to be affected by Parkinsonian motor symptoms.

Several additional mid-video frames including frames 19, 21, 22, 24, and 25 continued to contribute valuable signals through repeated eyebrow activations. These movements, even when subtle, encode a blend of voluntary and involuntary motor responses that correspond to motor control deficits commonly assessed in the MDS-UPDRS Part III examination. Late-frame contributions, captured across frames 27, 29, 31, 33, 35, and 43, provided further insights into residual motion patterns, particularly involving the right eye and eyebrow regions. These later cues reflect sustained or fading movement tendencies that help refine predictions by illustrating how facial motion evolves over time rather than relying solely on early or mid-frame behavior.

Taken together, the model’s emphasis on temporal distributions of eyebrow and eye landmarks demonstrates that dynamic periocular motion serves as a highly sensitive and physiologically meaningful marker for assessing Parkinson’s motor severity. By integrating both the density of key points and their changes across time, the model effectively captures fine-grained movement irregularities, enabling robust estimation of motor impairment levels in accordance with MDS-UPDRS Part III criteria.

## Discussion

The two regression analyses were conducted to describe the relationship between clinical symptom scores and disease severity rather than to diagnose Parkinson’s disease. Zero values for Hoehn & Yahr and MDS-UPDRS were assigned by clinical definition for non-Parkinson’s disease individuals, and therefore should not be interpreted as model-based predictions of disease absence or presence. Accordingly, the model is intended to provide explanatory insight into severity progression rather than diagnostic classification.

Beyond model performance, several important sources of bias were observed. Age-related bias was present due to differences in participants’ chronological age and age of Parkinson’s disease onset. Older participants naturally exhibited slower or less expressive facial movements, which sometimes resembled Parkinsonian hypomimia. This created the risk of misclassification, as the model may inadvertently learn age-related expressivity patterns rather than disease-specific signals. Similarly, younger-onset patients often retained stronger facial expressivity, potentially reducing predictive accuracy for severity and motor score estimation. This suggests that both age and onset variation may interact with model performance, warranting further quantitative investigation in future studies with larger cohorts. These performance drops are consistent with clinical experience that age affects facial muscle tone, eyebrow elevation, and smile intensity, independent of Parkinson’s disease progression. For example, natural age-related ptosis or reduced smile amplitude can mimic PD symptoms in older adults, while early-onset patients may show minimal facial rigidity despite clinically meaningful severity levels. These expert observations support the interpretation that part of the model error reflected in MAPE values of 38.36% and 45.33% may arise from age-related facial characteristics rather than Parkinsonian motor impairment alone.

This study has several limitations that should be acknowledged. First, the relatively small dataset of 40 participants may have impacted model stability, age-related generalizability, and susceptibility to demographic bias. Relatedly, feature importance was computed from a single model fit on the full dataset rather than averaged across cross-validation folds; fold-level stability analysis was not performed given the limited sample size, as further subdividing the data could reduce the reliability of such variance estimates. Second, the gender imbalance between groups (PD: 55% male; Control: 25% male) represents a potential confounding factor. Third, the assignment of zero scores to healthy controls for Hoehn & Yahr and MDS-UPDRS regression, while consistent with prior studies, represents a methodological simplification. Because these structural zeros account for half of the regression targets (20 of 40 participants) and are comparatively easy to predict once patients and controls are distinguished, the reported RMSE values may partly reflect this easier subset rather than the model’s ability to discriminate severity levels within the PD group specifically. A regression evaluated on PD participants only (e.g., via leave-one-out cross-validation, given the reduced sample size) would more directly test whether facial features carry severity-specific information, and is recommended for future work. Fourth, all recordings were collected under controlled clinical conditions with consistent lighting and camera positioning; performance in uncontrolled home environments remains to be validated. Fifth, medication status (ON/OFF state) was not systematically controlled during recording, which may have introduced variability in facial expressivity among PD participants. Future work should include a larger and more demographically balanced dataset, standardized medication protocols, and validation across multiple recording environments to enable robust age-adjusted modeling and real-world deployment. In addition, the feature engineering choices explored in this study, including video pre processing strategy, facial action scenario, and PCA dimensionality reduction, were evaluated empirically rather than through an exhaustive search, and alternative feature engineering strategies may further improve predictive performance. Furthermore, approximately 180 model-scenario-processing-PCA configurations were compared using the same 5-fold cross-validation splits, and the best-performing configuration for each task was reported as the headline result; this best-of-many selection procedure is optimistic relative to the true out-of-sample performance, particularly given the small test-fold size (approximately 8 participants per fold). This selection also applies within Tables 4, 6, and 8, where each reported value is itself the best result across all four Facial Action Scenarios for that processing method and PCA setting, compounding the optimism of the reported metrics. Future work should employ nested cross-validation, in which configuration selection and performance estimation are performed on separate data splits, to obtain an unbiased estimate of generalization performance. Relatedly, all performance metrics reported in this study represent point estimates averaged across the five cross-validation folds, without accompanying measures of dispersion (e.g., standard deviation or confidence intervals) or formal statistical testing between configurations. Given the modest fold size (approximately 8 participants per fold), comparisons described qualitatively in the Results (e.g., as showing stronger or weaker performance) should therefore be interpreted as descriptive trends rather than statistically confirmed differences.

## Conclusion

In this study, the performance of three main predictive models was evaluated and compared: a PD classification model to distinguish patients from controls (PD Classification), a Parkinson’s disease severity estimation model (Hoehn & Yahr), and a motor score estimation model (MDS-UPDRS Part III) using facial video data from 40 participants (20 PD patients and 20 healthy controls). The objectives were to investigate whether facial data alone can accurately predict disease presence and severity, and to analyze feature importance in each model to identify critical facial characteristics.

Overall, the XGBoost model combined with Frame Skipping and PCA achieved the best performance for PD classification during the smile scenario, while the Random Forest without PCA performed best for Hoehn & Yahr severity estimation, and the XGBoost model with 3-Frame Averaging and PCA performed best for MDS-UPDRS Part III estimation. Across models, lip and mouth corner movements were the most consistently important features for classification and Hoehn & Yahr severity, while eyebrow and eye movements were more important for MDS-UPDRS Part III estimation, highlighting the value of selecting appropriate facial action scenarios and dimensionality reduction methods for each task.

Facial-video-based assessment therefore offers a practical, lower-burden complement to conventional full-body clinical evaluation of Hoehn & Yahr severity.

This research can be extended to develop non-invasive Parkinson’s assessment tools suitable for real clinical environments, reducing evaluator burden. Integrating facial data with sensors or digital devices, such as wearables or full-body movement data, may enhance accuracy and enable long-term patient monitoring.

This study confirms that facial movements, particularly of the lips, mouth corners, eyebrows, and eyes, serve as critical representations of muscle behavior and Parkinson’s disease severity. These findings underscore the central role of feature engineering, that is, selecting appropriate video processing methods, dimensionality reduction via PCA, and suitable facial action scenarios, in enabling models to achieve accurate and reliable predictions. Using facial data alone is thus a promising approach for developing tools to assist in diagnosis and clinical assessment of Parkinson’s patients efficiently.

Future studies may integrate facial data with sensors or digital devices, such as wearables or full-body movement data, to enable long-term patient monitoring, and explore hybrid approaches combining OpenFace features with temporal deep learning architectures to capture long-range dependencies in facial motion sequences.

## References

[1] Kalia LV, Lang AE. Parkinson’s disease. Lancet. 2015;386(9996):896–912. doi: 10.1016/S0140-6736(14)61393-3 25904081

[2] Pearce JM. Aspects of the history of Parkinson’s disease. J Neurol Neurosurg Psychiatry. 1989;Suppl(Suppl):6–10. doi: 10.1136/jnnp.52.suppl.6 2666579 PMC1033302

[3] Carlsson A. Monoamine precursors and analogues. Pharmacol Ther B. 1975;1(3):381–92. doi: 10.1016/0306-039x(75)90044-6 772708

[4] Klein C, Westenberger A. Genetics of Parkinson’s disease. Cold Spring Harbor Perspectives in Medicine. 2012;2(1):a008888. doi: 10.1101/cshperspect.a008888 PMC3253033 22315721

[5] Goetz CG. The history of Parkinson’s disease: early clinical descriptions and neurological therapies. Cold Spring Harb Perspect Med. 2011;1(1):a008862. doi: 10.1101/cshperspect.a008862 22229124 PMC3234454

[6] Schulz JB, Falkenburger BH. Neuronal pathology in Parkinson’s disease. Journal of Neurology. 2004;251(Suppl 6):VI135–47.

[7] Ricciardi L, De Angelis A, Marsili L, Faiman I, Pradhan P, Pereira EA, et al. Hypomimia in Parkinson’s disease: an axial sign responsive to levodopa. Eur J Neurol. 2020;27(12):2422–9. doi: 10.1111/ene.14452 32702196

[8] Hoehn MM, Yahr MD. Parkinsonism: onset, progression and mortality. Neurology. 1967;17(5):427–42. doi: 10.1212/wnl.17.5.427 6067254

[9] Goetz CG, Tilley BC, Shaftman SR, Stebbins GT, Fahn S, Martinez-Martin P. Movement Disorder Society-sponsored revision of the Unified Parkinson’s Disease Rating Scale (MDS-UPDRS). Movement Disorders. 2008;23(15):2129–70. 10.1002/mds.22340 19025984

[10] Jackson H, Anzures-Cabrera J, Taylor KI, Pagano G, PASADENA Investigators, Prasinezumab Study Group. Hoehn and Yahr Stage and Striatal Dat-SPECT Uptake Are Predictors of Parkinson’s Disease Motor Progression. Front Neurosci. 2021;15:765765. doi: 10.3389/fnins.2021.765765 34966256 PMC8711238

[11] Jaratsaeng S, Techasen A, Kasemsap N, Intharah T. PDFace: Parkinson’s Disease Classification Via Movement Analysis of Video From Mobile Camera. In: 2026 18th International Conference on Knowledge and Smart Technology (KST), 2026. 59–64. 10.1109/kst67832.2026.11432360

[12] Baltrušaitis T, Zadeh A, Lim YC, Morency LP. OpenFace 2.0: Facial Behavior Analysis Toolkit. In: 2018 13th IEEE International Conference on Automatic Face and Gesture Recognition (FG 2018); 2018. p. 59–66. 10.1109/FG.2018.00019

[13] King D. Dlib-ml: A Machine Learning Toolkit. Journal of Machine Learning Research. 2009;10:1755–8.

[14] Baltrusaitis T, Robinson P, Morency L-P. OpenFace: An open source facial behavior analysis toolkit. In: 2016 IEEE Winter Conference on Applications of Computer Vision (WACV), 2016. 1–10. 10.1109/wacv.2016.7477553

[15] Bandini A, Orlandi S, Escalante HJ, Giovannelli F, Cincotta M, Reyes-Garcia CA, et al. Analysis of facial expressions in parkinson’s disease through video-based automatic methods. J Neurosci Methods. 2017;281:7–20. doi: 10.1016/j.jneumeth.2017.02.006 28223023

[16] Rajnoha M, Mekyska J, Burget R, Eliasova I, Kostalova M, Rektorova I. Towards Identification of Hypomimia in Parkinson’s Disease Based on Face Recognition Methods. In: 2018 10th International Congress on Ultra Modern Telecommunications and Control Systems and Workshops (ICUMT), 2018. 1–4. 10.1109/icumt.2018.8631249

[17] Hou X, Qin S, Su J. Visual Detection of Parkinson’s Disease via Facial Features Recognition. Lecture Notes in Electrical Engineering. Springer Singapore. 2021. p. 249–57. doi: 10.1007/978-981-16-6372-7_29

[18] Ma LY, Tian Y, Pan CR, Chen ZL, Ling Y, Ren K. Motor progression in early-stage Parkinson’s disease: Prediction using regression and machine learning models. Frontiers in Aging Neuroscience. 2021;12:627199. 10.3389/fnagi.2020.627199 PMC7868416 33568988

[19] Adnan T, Islam MS, Rahman W, Lee S, Tithi SD, Noshin K, et al. Unmasking Parkinson’s Disease with Smile: An AI-enabled Screening Framework. arXiv preprint. 2023. arXiv:2308.02588.

[20] Duan S, Dadashzadeh A, Whone A, Mirmehdi M. QAFE-Net: Quality Assessment of Facial Expressions with Landmark Heatmaps. In: Proceedings of the IEEE/CVF Winter Conference on Applications of Computer Vision (WACV); 2024. p. 6308–17.

[21] Islam MS, Adnan T, Freyberg J, Lee S, Abdelkader A, Pawlik M, et al. Accessible, At-Home Detection of Parkinson’s Disease via Multi-task Video Analysis. arXiv preprint. 2024. arXiv:2406.14856.

[22] di Biase L, Pecoraro PM, Bugamelli F. AI Video Analysis in Parkinson’s Disease: A Systematic Review of the Most Accurate Computer Vision Tools for Diagnosis, Symptom Monitoring, and Therapy Management. Sensors (Basel). 2025;25(20):6373. doi: 10.3390/s25206373 41157427 PMC12568243

[23] Colautti L, Casella M, Robba M, Marocco D, Ponticorvo M, Iannello P. Predicting Cognitive Decline in Parkinson’s Disease Using Artificial Neural Networks: An Explainable AI Approach. Brain Sciences. 2025;15(8):782. doi: 10.3390/brainsci15080782 PMC12384829 40867115

[24] Jolliffe IT. Principal Component Analysis. 2nd ed. Springer. 2002.

[25] Cortes C, Vapnik V. Support-Vector Networks. Machine Learning. 1995;20(3):273–97. doi: 10.1023/a:1022627411411

[26] Breiman L. Random Forests. Machine Learning. 2001;45(1):5–32. doi: 10.1023/A:1010933404324

[27] Chen T, Guestrin C. XGBoost: A scalable tree boosting system. In: Proceedings of the 22nd ACM SIGKDD International Conference on Knowledge Discovery and Data Mining; 2016. p. 785–94.

[28] Kohavi R. A study of cross-validation and bootstrap for accuracy estimation and model selection. In: Proceedings of the 14th International Joint Conference on Artificial Intelligence; 1995. p. 1137–45.

[29] Jaratsaeng S, Techasen A, Kasemsap N, Intharah T. Parkinson’s disease assessment using mobile phone camera. Harvard Dataverse. 2026. doi: 10.7910/DVN/CT34N0 PMC13596831 42771663

[30] Pedregosa F, Varoquaux G, Gramfort A, Michel V, Thirion B, Grisel O, et al. Scikit-learn: Machine Learning in Python. Journal of Machine Learning Research. 2011;12:2825–30.

[31] Goetz CG, Poewe W, Rascol O, Sampaio C, Stebbins GT, Counsell C, et al. Movement Disorder Society Task Force report on the Hoehn and Yahr staging scale: status and recommendations. Movement Disorders. 2004;19(9):1020–8. doi: 10.1002/mds.20213 15372591

[32] Horváth K, Aschermann Z, Ács P, Deli G, Janszky J, Komoly S, et al. Minimal clinically important difference on the Motor Examination part of MDS-UPDRS. Parkinsonism Relat Disord. 2015;21(12):1421–6. doi: 10.1016/j.parkreldis.2015.10.006 26578041

