# Supplementary material for: Parkinson’s disease assessment with selfie video via feature engineering and explainable AI
Source: PLoS One. 2026 Sep 22;21(9):e0357902. doi: 10.1371/journal.pone.0357902 (PMC13596831; doi:10.1371/journal.pone.0357902)
Supplement: Supplementary file 1 [file pone.0357902.s002.pdf]

Response Letter PlosONE

Parkinson's Disease Assessment with Selfie Video via Feature Engineering and Explainable AI

Response to Editor's Requirements:

Comments	Response
1. Please ensure that your manuscript meets PLOS ONE's style requirements, including those for file naming. The PLOS ONE style templates.	We thank the editor for this reminder. We have carefully reviewed our manuscript against the PLOS ONE style templates provided and made the following corrections to ensure full compliance:<br>1. **File naming and format for figures:** All figures have been renamed following the PLOS ONE convention (e.g., "Fig1", "Fig2") and saved in an acceptable file format (.tif) for submission. Note that the LaTeX source file references the figures in .png format, as our compiler (Overleaf/pdfLaTeX) does not support direct compilation with .tif files; however, the .tif versions of all figures have been prepared separately and are

Comments	Response
	included with this submission as the actual figure files to be used in production.<br>2. **Figure and table captions:** All captions have been reformatted so that "FigN." / "TableN." and the accompanying title are presented in bold, consistent with the formatting guidelines.<br>3. **Title, author, and affiliation page:** We removed postal/ZIP codes from all author affiliations and added the corresponding author's initials in parentheses after the email address, in line with the formatting sample provided.
2. Please note that PLOS One has specific guidelines on code sharing for submissions in which author-generated code underpins the findings in the manuscript. In these cases, we expect all author-generated code to be made available without restrictions upon publication of the work. Please review our guidelines at https://journals.plos.org/plosone/s/materials-and-software-sharing#loc-sharing-code and ensure that your code is shared in a way that follows best practice and facilitates reproducibility and reuse.	We thank the editor for highlighting PLOS ONE's code sharing policy. We confirm that all author-generated code underlying the findings of this manuscript has been made publicly available without restriction, in accordance with the guidelines provided. Specifically, as detailed in the *Data and Code Availability* section

Comments	Response
	(page 27). Both repositories are public, include a README file, and are licensed under the MIT License to facilitate reproducibility and reuse. We believe this fully satisfies PLOS ONE's code sharing requirements.
3. We note that Figures 1 and 2 include images of a participant in the study. As per the PLOS ONE policy (http://journals.plos.org/plosone/s/submission-guidelines#loc-human-subjects-research) on papers that include identifying, or potentially identifying, information, the individual(s) or parent(s)/guardian(s) must be informed of the terms of the PLOS open-access (CC-BY) license and provide specific permission for publication of these details under the terms of this license. Please download the Consent Form for Publication in a PLOS Journal (http://journals.plos.org/plosone/s/file?id=8ce6/plos-consent-form-english.pdf). The signed consent form should not be submitted with the manuscript, but should be securely filed in the individual's case notes. Please amend the methods section and ethics statement of the manuscript to explicitly state that the patient/participant has provided consent for publication: "The individual in this manuscript has given written informed consent (as outlined in PLOS consent form) to publish these case details".	We thank the editor for this important reminder regarding PLOS ONE's policy on identifying information. We confirm that the individual shown in Figs 1 and 2 is one of the co-authors of this manuscript, and written informed consent for publication has been obtained using the official PLOS Consent Form. In accordance with PLOS ONE policy, the signed consent form has not been submitted with the manuscript but is securely filed with the individual's records.<br><br>We have amended the Ethics statement section of the revised manuscript (page 7).

Response to the Comments of Reviewer I:

**Comments**	**Response**
1. Broadening the scope of XAI in Parkinson's Disease. In the Related Work section, the discussion on Explainable AI is primarily centered around computer vision and landmark detection. The reviewer suggests briefly expanding the review to include how XAI is being applied to other clinical dimensions of the disease, such as non-motor symptoms, and recommends citing Colautti et al. (2025), Predicting Cognitive Decline in Parkinson's Disease Using Artificial Neural Networks: An Explainable AI Approach, Brain Sciences, 15(8), 782.	We thank the reviewer for this valuable suggestion. We have expanded the Related Work section (page 5) to broaden the discussion of Explainable AI beyond computer vision and facial landmark-based approaches. Specifically, we added a discussion of Colautti et al. [22], who applied artificial neural networks combined with explainable AI techniques to predict cognitive decline in Parkinson's disease patients, demonstrating that XAI can provide interpretable insights into non-motor clinical dimensions of the disease. We note that this broader application of XAI across both motor and non-motor dimensions underscores its growing role as a critical tool for enhancing the transparency and clinical relevance of machine learning-based assessment methods.<br><br>In addition, in response to this and related comments, we have further strengthened the Explainable AI component of this work by adding a complete set of mathematical formulations (Equations 1–7) detailing how feature importance is computed for both the non-PCA and PCA-based conditions, together with an expanded explanation of the PCA back-mapping procedure and the elbow-point method used to select influential

Comments	Response
	features. These additions provide a more rigorous and transparent methodological foundation for the XAI analysis, reinforcing its role as a central contribution of this study rather than a supplementary component.
2. Given the relatively small sample size of 40 participants, machine learning models can sometimes yield unstable feature importances due to slight variations in the training data. Did the authors evaluate the variance or stability of the extracted important features across the 5 folds of the cross-validation? A brief note on this in the Results or Discussion section would strengthen the reliability of the XAI findings.	We thank the reviewer for raising this important point regarding the stability of feature importance estimates given our limited sample size.<br><br>We would like to clarify that feature importance in this study was computed from a single model fit using the full dataset, rather than averaged across the 5 cross-validation folds. The 5-fold cross-validation procedure described in the Methods was used specifically to evaluate predictive performance (accuracy, precision, recall, f1-score, RMSE, and MAPE), not to assess feature importance stability.<br><br>Given the relatively small sample size (n = 40), we did not perform a fold-level feature importance stability analysis, as further subdividing the data would leave too few samples per fold to yield statistically reliable variance estimates. We acknowledge this as a limitation of the current study and have revised the Limitations paragraph of the Discussion section (page 25-26) to accurately reflect that feature importance was derived from a single fit on the full dataset. We agree that evaluating feature importance stability

Comments	Response
	across folds would strengthen the reliability of the XAI findings, and we have noted this as a direction for future work with larger cohorts.
3. Ensure consistency in formatting throughout the text. In some sections, terms like "XGBoost Model" or "Random Forest Model" use varying capitalization, and "OpenFace 2.0" is occasionally written with a space ("Open Face 2.0" on line 118).	We thank the reviewer for pointing out these formatting inconsistencies. We have carefully reviewed the entire manuscript and standardized capitalization and spelling throughout, including model and tool names (e.g., Random Forest, XGBoost, OpenFace 2.0), as well as recurring terms such as 'Parkinson's disease,' 'machine learning,' 'video processing,' 'Facial Action Scenario,' and 'F1-score.

Response to the Comments of Reviewer II:

Comments	Response
1. The reviewer suggests clarifying the manuscript title as "Parkinson's Disease Prediction Techniques Based Artificial Intelligence Video Processing".	We thank the reviewer for this suggestion regarding the manuscript title. While we appreciate the reviewer's proposed title, we have instead revised the title to: **"Parkinson's Disease Assessment with Selfie Video via Feature Engineering and Explainable AI"**<br><br>We chose this revised title because it more accurately reflects the core contributions and workflow of the study: (1) the use of selfie (smartphone-captured) video rather than a

Comments	Response
	general video-processing framework, (2) the central role of feature engineering in our five preprocessing strategies and PCA-based dimensionality reduction, and (3) the emphasis on Explainable AI, which is a key contribution of this work distinguishing it from prior facial-video-based PD studies. We believe this title better captures the specific methodological focus of the manuscript while still directly conveying that the study concerns AI-based, video-derived assessment of Parkinson's disease, which we understand to be the reviewer's underlying concern.
2. In the introduction section, the reviewer suggests moving the paragraph contents starting from line 31 and ending at line 39 into the related work section.	We thank the reviewer for this suggestion. The relevant paragraph has been moved to the Related Work section (page 3) as recommended.
3. In the related work section, the reviewer suggests moving the paragraph contents starting from line 99 and ending at line 127 into the introduction section.	We thank the reviewer for this suggestion. The relevant paragraph has been moved to the Introduction section (page 2) as recommended.
4. Rearrangement of overall abbreviations over the manuscript to eliminate repetitions, such as the repetition of Parkinson's disease (PD) at the first line of the abstract and then repeated at the related work section line 185, and at the methodology section line 220.	We thank the reviewer for this observation. We have reviewed the entire manuscript and removed redundant re-definitions of abbreviations, including "Parkinson's disease (PD)," which was previously repeated in the Abstract, Related Work (line 185), and Methodology (line 220) sections. We also reviewed and corrected other repeated or redundantly re-explained abbreviations

Comments	Response
	throughout the manuscript to ensure each term is defined only at its first occurrence.
5. In the related work section, the reviewer suggests moving the contents starting from line 215 and ending at line 217 into the conclusion section.	We thank the reviewer for this suggestion. The relevant content has been moved to the Conclusion section (pages 26–27) as recommended. We note that this relocation is connected to Reviewer 2's Comment 8.1 regarding future work, as the moved content pertains to the broader clinical relevance and future applicability of Explainable AI, which now fits more coherently alongside the future work discussion in the Conclusion.
6. Authors have to avoid using pronouns, and use passive voice instead.	We thank the reviewer for this comment. We have carefully reviewed the entire manuscript and revised all instances where first-person pronouns (e.g., "we") were used as the subject, converting them to passive voice construction throughout.
7. Basically, the manuscript has to be proofread due to many occurrences of grammar errors.	We thank the reviewer for this comment. We have thoroughly proofread the entire manuscript from the Abstract through the References, correcting grammatical errors, missing punctuation, incomplete sentences, and redundant or repeated content throughout.
8. Large repetition in the discussion section and conclusion section; the author would reduce repetitions by clarifying these two sections carefully.	We thank the reviewer for this comment. We have carefully revised both the Discussion and Conclusion sections to reduce redundancy between them.<br><br>First, regarding the future work content, we

Comments	Response
	removed a sentence recommending larger and more diverse patient cohorts, as this point was already addressed in the Discussion's Limitations paragraph. We retained the future work statement on integrating facial data with sensors or wearable devices for long-term monitoring, as this represents a distinct idea not covered elsewhere. We also merged a previously isolated sentence on hybrid deep learning approaches into the same future work paragraph, rather than leaving it as a standalone closing statement.<br><br>Second, the Conclusion was substantially condensed. The lengthy paragraph summarizing detailed numerical results for each predictive task (classification, severity estimation, and motor score estimation) was shortened into a single, concise paragraph that highlights only the best-performing model for each task, without repeating specific metric values already reported in the Results and interpreted in the Discussion.<br><br>Third, we identified that the opening paragraph of the Discussion and the clinical perspective paragraph in the Conclusion conveyed a similar underlying argument. We removed the lengthy explanatory reasoning from the Conclusion and retained only a brief, single-sentence summary, directing readers back to the Discussion for the full

Comments	Response
	clinical rationale.<br><br>Collectively, these revisions have substantially reduced repetition while preserving all essential findings and interpretations.
