# Supplementary material for: Parkinson’s disease assessment with selfie video via feature engineering and explainable AI
Source: PLoS One. 2026 Sep 22;21(9):e0357902. doi: 10.1371/journal.pone.0357902 (PMC13596831; doi:10.1371/journal.pone.0357902)
Supplement: Peer review history

# Peer review: Decision Letter 0

**DOI:** 10.1371/journal.pone.0357902.r001

**Authors:** Ali Al-Naji

9 Aug 2026

Dear Dr. Intharah,

Thank you for submitting your manuscript to PLOS ONE. After careful consideration, we feel that it has merit but does not fully meet PLOS ONE’s publication criteria as it currently stands. Therefore, we invite you to submit a revised version of the manuscript that addresses the points raised during the review process.

Please submit your revised manuscript by Sep 23 2026 11:59PM. If you will need more time than this to complete your revisions, please reply to this message or contact the journal office at plosone@plos.org. When you're ready to submit your revision, log on to https://www.editorialmanager.com/pone/ and select the 'Submissions Needing Revision' folder to locate your manuscript file.

- A letter that responds to each point raised by the academic editor and reviewer(s). You should upload this letter as a separate file labeled 'Response to Reviewers'.
- A marked-up copy of your manuscript that highlights changes made to the original version. You should upload this as a separate file labeled 'Revised Manuscript with Track Changes'.
- An unmarked version of your revised paper without tracked changes. You should upload this as a separate file labeled 'Manuscript'.

If you would like to make changes to your financial disclosure, please include your updated statement in your cover letter. Guidelines for resubmitting your figure files are available below the reviewer comments at the end of this letter.

If applicable, we recommend that you deposit your laboratory protocols in protocols.io to enhance the reproducibility of your results. Protocols.io assigns your protocol its own identifier (DOI) so that it can be cited independently in the future. For instructions see: https://journals.plos.org/plosone/s/submission-guidelines#loc-laboratory-protocols. Additionally, PLOS ONE offers an option for publishing peer-reviewed Lab Protocol articles, which describe protocols hosted on protocols.io. Read more information on sharing protocols at https://plos.org/protocols?utm_medium=editorial-email&utm_source=authorletters&utm_campaign=protocols.

As the corresponding author, your ORCID iD is verified in the submission system and will appear in the published article. PLOS supports the use of ORCID, and we encourage all coauthors to register for an ORCID iD and use it as well. Please encourage your coauthors to verify their ORCID iD within the submission system before final acceptance, as unverified ORCID iDs will not appear in the published article. *Only*  the individual author can complete the verification step; PLOS staff *cannot*  verify ORCID iDs on behalf of authors.

We look forward to receiving your revised manuscript.

Kind regards,

Ali Al-Naji

Academic Editor

PLOS One

Journal requirements:

When submitting your revision, we need you to address these additional requirements.

1. Please ensure that your manuscript meets PLOS ONE's style requirements, including those for file naming. The PLOS ONE style templates can be found at

https://journals.plos.org/plosone/s/file?id=wjVg/PLOSOne_formatting_sample_main_body.pdf and

https://journals.plos.org/plosone/s/file?id=ba62/PLOSOne_formatting_sample_title_authors_affiliations.pdf

2. Please note that PLOS One has specific guidelines on code sharing for submissions in which author-generated code underpins the findings in the manuscript. In these cases, we expect all author-generated code to be made available without restrictions upon publication of the work. Please review our guidelines at https://journals.plos.org/plosone/s/materials-and-software-sharing#loc-sharing-code and ensure that your code is shared in a way that follows best practice and facilitates reproducibility and reuse.

3. We note that Figures 1 and 2 include images of a participant in the study.

As per the PLOS ONE policy (http://journals.plos.org/plosone/s/submission-guidelines#loc-human-subjects-research) on papers that include identifying, or potentially identifying, information, the individual(s) or parent(s)/guardian(s) must be informed of the terms of the PLOS open-access (CC-BY) license and provide specific permission for publication of these details under the terms of this license. Please download the Consent Form for Publication in a PLOS Journal (http://journals.plos.org/plosone/s/file?id=8ce6/plos-consent-form-english.pdf). The signed consent form should not be submitted with the manuscript, but should be securely filed in the individual's case notes. Please amend the methods section and ethics statement of the manuscript to explicitly state that the patient/participant has provided consent for publication: “The individual in this manuscript has given written informed consent (as outlined in PLOS consent form) to publish these case details”.

If you are unable to obtain consent from the subject of the photograph, you will need to remove the figure and any other textual identifying information or case descriptions for this individual.

If the reviewer comments include a recommendation to cite specific previously published works, please review and evaluate these publications to determine whether they are relevant and should be cited. There is no requirement to cite these works unless the editor has indicated otherwise.

[Note: HTML markup is below. Please do not edit.]

Reviewers' comments:

Reviewer's Responses to Questions

**Comments to the Author**

1. Is the manuscript technically sound, and do the data support the conclusions?

Reviewer #1: Yes

Reviewer #2: Yes

**********

2. Has the statistical analysis been performed appropriately and rigorously?

Reviewer #1: Yes

Reviewer #2: Yes

**********

3. Have the authors made all data underlying the findings in their manuscript fully available?

The PLOS Data policy

Reviewer #1: Yes

Reviewer #2: Yes

**********

4. Is the manuscript presented in an intelligible fashion and written in standard English?

Reviewer #1: Yes

Reviewer #2: Yes

**********

Reviewer #1: The manuscript investigates a highly relevant and impactful topic: the use of non-invasive, accessible smartphone selfie videos combined with machine learning to assess Parkinson’s Disease (PD). The authors attempt a multi-faceted task, analyzing facial dynamics (hypomimia) not only for binary PD classification but also for predicting disease severity via the Hoehn & Yahr scale and estimating motor function through MDS-UPDRS Part III scores.

The study presents a comprehensive technical framework. The inclusion of Explainable AI (XAI) methods to map feature importance back to specific facial structures (such as the lips and eyebrows) provides interpretability to these "black box" algorithms.

Overall, this is a well-structured study that fits well within the scope of PLOS ONE.

I suggest to consider the following points:

- broadening the Scope of XAI in Parkinson’s Disease: in the Related Work section, the discussion on Explainable AI is primarily centered around computer vision and landmark detection. To provide readers with a more holistic view of how interpretable AI is transforming Parkinson's management, the authors should briefly expand their review to include how XAI is being applied to other clinical dimensions of the disease, such as non-motor symptoms.

Recommendation: I suggest referencing recent advancements where explainable architectures have been successfully leveraged to predict other complex progressive aspects of PD. For instance, you could cite:

Colautti, L., Casella, M., Robba, M., Marocco, D., Ponticorvo, M., Iannello, P., ... & CPP Integrated Parkinson’s Database. (2025). Predicting Cognitive Decline in Parkinson’s Disease Using Artificial Neural Networks: An Explainable AI Approach. Brain Sciences, 15(8), 782.

- given the relatively small sample size of 40 participants, machine learning models can sometimes yield unstable feature importances due to slight variations in the training data. Did the authors evaluate the variance or stability of the extracted important features across the 5 folds of the cross-validation? A brief note on this in the Results or Discussion section would strengthen the reliability of the XAI findings.

Some minor points:

- ensure consistency in formatting throughout the text. In some sections, terms like "XGBoost Model" or "Random Forest Model" use varying capitalization, and "OpenFace 2.0" is occasionally written with a space ("Open Face 2.0" on line 118)

Reviewer #2: The manuscript well organized by considering standard research methodologies, however the research hypothesis with wide variation parameters that leaded not promises outcomes. The reviewer would nominate the manuscript to be accepted with minor corrections by considering the followings:

1. Reviewer suggests to clarify the manuscript title as

“Parkinson’s Disease Prediction Techniques Based Artificial Intelligence Video Processing”

2. In an introduction section, the reviewer suggests to move the paragraph contents start from line 31 and ended at line 39 into related work section.

3. In a related work section, the reviewer suggests to move the paragraph contents start from line 99 and ended at line 127 into introduction section.

4. Rearrangement of overall abbreviations over the manuscript to eliminate repetitions, such a repetition of Parkinson’s disease (PD) at the first line of an abstract and then repeated times at the related work section line 185, at methodology section line 220.

5. In a related work section, the reviewer suggests to move the contents start from line 215 and ended at line 217 into conclusion section.

6. Authors have to avoid using pronoun, and using passive voice instead.

7. Basically, the manuscript has to be proof reading due to many occurrences of grammar errors.

8. Large repetition in discussion section and conclusion section, author would reduce repetitions by clarifying these two sections carefully.

**********

what does this mean?). If published, this will include your full peer review and any attached files.

If you choose “no”, your identity will remain anonymous but your review may still be made public.

**Do you want your identity to be public for this peer review?**  For information about this choice, including consent withdrawal, please see our Privacy Policy

Reviewer #1: No

Reviewer #2: **Yes:** Aws Alazawi

Assistant Professor PhD in Biomedical Signal Processing

Department of Medical Instrumentation Engineering Techniques

Technical College of Electrical Engineering

Middle Technical University

**********

[NOTE: If reviewer comments were submitted as an attachment file, they will be attached to this email and accessible via the submission site. Please log into your account, locate the manuscript record, and check for the action link "View Attachments". If this link does not appear, there are no attachment files.]

To ensure your figures meet our technical requirements, please review our figure guidelines: https://journals.plos.org/plosone/s/figures

You may also use PLOS’s free figure tool, NAAS, to help you prepare publication quality figures: https://journals.plos.org/plosone/s/figures#loc-tools-for-figure-preparation.

NAAS will assess whether your figures meet our technical requirements by comparing each figure against our figure specifications.

# Author comment: Author response to Decision Letter 1

**DOI:** 10.1371/journal.pone.0357902.r002

20 Aug 2026

The response to reviewers file is attached.

Attachment Submitted filename: Response-to-Review-Comments-PlosONE.pdf

# Editor report: Decision Letter 1

**DOI:** 10.1371/journal.pone.0357902.r003

**Authors:** Ali Al-Naji

24 Aug 2026

Parkinson’s Disease Assessment with Selfie Video via Feature Engineering and Explainable AI

PONE-D-26-17752R1

Dear Dr. Intharah,

We’re pleased to inform you that your manuscript has been judged scientifically suitable for publication and will be formally accepted for publication once it meets all outstanding technical requirements.

Within one week, you’ll receive an e-mail detailing the required amendments. When these have been addressed, you’ll receive a formal acceptance letter and your manuscript will be scheduled for publication.

An invoice will be generated when your article is formally accepted. Please note, if your institution has a publishing partnership with PLOS and your article meets the relevant criteria, all or part of your publication costs will be covered. Please make sure your user information is up-to-date by logging into Editorial Manager at Editorial Manager® and clicking the ‘Update My Information' link at the top of the page. For questions related to billing, please contact billing support.

If your institution or institutions have a press office, please notify them about your upcoming paper to help maximize its impact. If they’ll be preparing press materials, please inform our press team as soon as possible -- no later than 48 hours after receiving the formal acceptance. Your manuscript will remain under strict press embargo until 2 pm Eastern Time on the date of publication. For more information, please contact onepress@plos.org.

Kind regards,

Ali Al-Naji

Academic Editor

PLOS One

Additional Editor Comments (optional):

Reviewers' comments:

# Editor report: Acceptance letter

**DOI:** 10.1371/journal.pone.0357902.r004

**Authors:** Ali Al-Naji

PONE-D-26-17752R1

PLOS One

Dear Dr. Intharah,

I'm pleased to inform you that your manuscript has been deemed suitable for publication in PLOS One. Congratulations! Your manuscript is now being handed over to our production team.

At this stage, our production department will prepare your paper for publication. This includes ensuring the following:

* All references, tables, and figures are properly cited

* All relevant supporting information is included in the manuscript submission,

* There are no issues that prevent the paper from being properly typeset

You will receive further instructions from the production team, including instructions on how to review your proof when it is ready. Please keep in mind that we are working through a large volume of accepted articles, so please give us a few days to review your paper and let you know the next and final steps.

Lastly, if your institution or institutions have a press office, please let them know about your upcoming paper now to help maximize its impact. If they'll be preparing press materials, please inform our press team within the next 48 hours. Your manuscript will remain under strict press embargo until 2 pm Eastern Time on the date of publication. For more information, please contact onepress@plos.org.

You will receive an invoice from PLOS for your publication fee after your manuscript has reached the completed accept phase. If you receive an email requesting payment before acceptance or for any other service, this may be a phishing scheme. Learn how to identify phishing emails and protect your accounts at https://explore.plos.org/phishing.

If we can help with anything else, please email us at customercare@plos.org.

Thank you for submitting your work to PLOS One and supporting open access.

Kind regards,

PLOS One Editorial Office Staff

on behalf of

Dr. Ali Al-Naji

Academic Editor

PLOS One
